# Proximity labelling of internalizing influenza A viruses reveals a role for neogenin in virus uptake

**DOI:** 10.1371/journal.ppat.1013338

**Published:** 2025-07-07

**Authors:** Milagros Sempere Borau, Victor G. Gisbert, Josephine von Kempis, Laura M. Arroyo-Fernández, Samira Schiefer, David Alsteens, Silke Stertz

**Affiliations:** 1 Institute of Medical Virology, University of Zurich, Zurich, Switzerland; 2 Louvain Institute of Biomolecular Science and Technology (LIBST), UCLouvain, Louvain, Belgium; Icahn School of Medicine at Mount Sinai, UNITED STATES OF AMERICA

## Abstract

Influenza A virus (IAV) is a respiratory pathogen of global concern. Entry of most IAVs is mediated by binding of viral hemagglutinin to cellular sialic acid, facilitating virus attachment. A subsequent interaction with a surface receptor(s) triggers viral uptake. Although multiple host factors involved in viral entry are known, the identity of these receptors remains unclear. Here, we utilized proximity labelling to acquire the interactome of epsin 1, an adaptor protein utilized by IAV for clathrin-mediated endocytosis, during virus internalization to identify them. We uncover neogenin (Neo1), a member of the immunoglobulin superfamily expressed in primary human airway cultures, as a potential epsin 1 interactor and virus receptor candidate. Knockdown of Neo1 led to a reduction in replication of H1N1, H2N2 and H5N1 IAVs in primary and immortalized lung cells. Moreover, human recombinant Neo1 was found to bind IAV with a K_D_ of 21 ± 14 nM by atomic force microscopy and Neo1 could co-localize with incoming IAV at early times post-infection, as well as affect viral entry. As Neo1 can interact with IAV and its depletion impairs IAV entry, this study reveals its potential as an IAV internalization receptor.

## Author summary

The majority of influenza A viruses (IAVs) enter host cells through glycan-dependent attachment at the cell surface and subsequent endocytosis following the encounter with a signalling receptor. However, an indispensable IAV internalization receptor has not been identified thus far and which host proteins can exert this function is unclear. In this study, we performed proximity labelling during virus uptake to identify proteins surrounding epsin 1, which is an adaptor protein necessary for clathrin-mediated endocytosis of IAV, with the goal of discovering novel internalization receptors. We discovered Neo1 as receptor candidate and assessed whether it could act as such. The depletion of Neo1 expression reduced infection of reporter IAVs of multiple subtypes (H1N1, H2N2 and H5N1) as well as authentic virus and its proviral function could be confirmed in primary lung fibroblast cells. We also showed that Neo1 can interact with IAV with high affinity, co-localize with incoming virus at early infection stages and affect viral entry, which is reduced in its absence. Overall, these data evidence the potential of Neo1 as an IAV internalization receptor and contribute to our understanding of virus entry.

## Introduction

Influenza A virus (IAV) is a respiratory pathogen that poses a significant challenge to global health. Antigenic drift results in seasonal human IAV infections that amount to 3–5 million cases of severe illness and 290 000–650 000 deaths annually [1]. Due to their reassortment ability and continued circulation in animal reservoirs, IAVs have caused four pandemics with substantial morbidity and mortality [2–4]. Thus, it is essential to comprehensively understand IAV biology in order to combat it through effective antiviral design.

Entry into host cells is an essential step of the IAV replication cycle that remains poorly understood. The majority of IAVs, subtypes H1-16, attach to cells through multivalent binding of viral hemagglutinin to terminal sialic acid on cell surface glycoproteins and glycolipids [2,5,6]. Viral attachment and sialic acid binding preference have important implications for IAV entry and tropism, which have been described extensively [6–8]. However, binding to sialic acid does not always confer virus internalization and sialic acid moieties do not possess signalling capabilities, meaning that they would not be sufficient to trigger IAV uptake [9–11]. Consequently, it has been speculated that interactions with a cellular receptor(s) may be required to specifically trigger IAV uptake [9,12–14] and multiple studies have been conducted to identify the host factors involved [12,13,15–18]. Amongst them, the host factors nucleolin, EGFR, IGDCC4, CaV1.2, mGluR2 and FFAR2 have been shown to bind IAV HA and affect virus internalization, yet their individual inhibition [13,16], knockout [12,15] or depletion through RNAi [13,16,17] did not abolish virus internalization nor confer a change in tropism [12]. Moreover, their effect on virus internalization varied in extent according to the IAV strain or subtype used [17]. The extent of the IAV internalization receptor repertoire is thus unclear and we hypothesized that there are more receptors to be identified.

Following engagement with an internalization receptor, IAV can be internalized through multiple endocytic routes, including clathrin-mediated endocytosis (CME) [19–22], clathrin-independent endocytosis (CIE) [21,23] or macropinocytosis [24,25]. Viral uptake through CME, a major IAV entry route [20], is reliant on the adaptor protein epsin 1 [19]. Knockdown of epsin 1 or overexpression of a mutant lacking its ubiquitin interacting motif significantly decreased CME of IAV and instead re-routed uptake through CIE [19]. As epsin 1 can interact with ubiquitinated receptors to facilitate their uptake [26,27] we hypothesized that epsin 1 may be in the vicinity of internalization receptors upon virus infection.

Here, we performed proximity labelling to identify proteins in the vicinity of epsin 1 during virus infection as a means to uncover IAV internalization receptors. Within the epsin 1 interactome, we found proteins associated with wild type epsin 1 function and location, known interactors and host factors involved in IAV infection, validating our approach. We identified neogenin (Neo1), a member of the immunoglobulin superfamily expressed on human primary airway cells, as an IAV receptor candidate. Knockdown of Neo1 in A549 cells decreased replication of IAVs of multiple subtypes and recombinant human Neo1 could interact with IAVs as determined by atomic force microscopy. Lastly, Neo1 could co-localize with incoming IAV in non-permeabilized and permeabilized samples at early times post-infection and its depletion reduced viral entry, highlighting its potential as a virus receptor.

## Results

### Acquisition of the epsin 1 interactome upon virus infection

**Fig 1:**
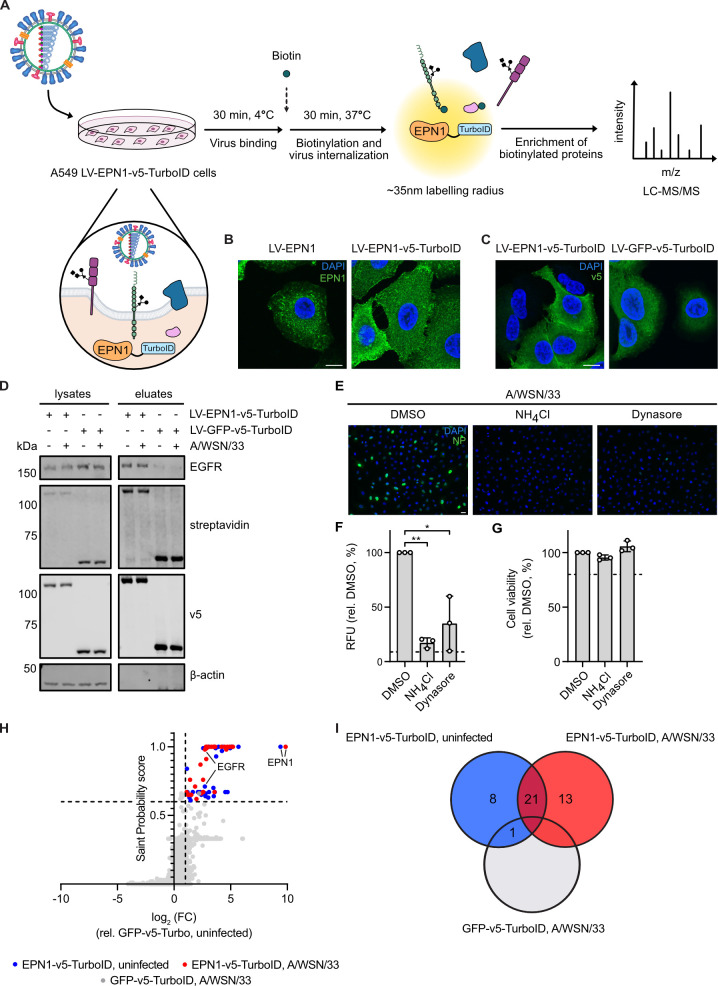
Acquisition of the epsin 1 interactome upon IAV infection. **A)** Schematic depicting the experimental workflow to obtain the epsin 1 interactome upon IAV infection in A549 cells stably expressing epsin 1 fused to TurboID. Virus schematic adapted from [29]. **B)** A549 cells were transduced with lentiviruses encoding epsin 1 (LV-EPN1) or epsin 1 tagged with v5 and TurboID (LV-EPN1-v5-TurboID). The expression of epsin 1 was evaluated via confocal microscopy. The images depict nuclei (DAPI, blue) and epsin 1 (EPN1, green). **C)** A549 cells stably expressing GFP tagged with v5 and Turbo ID (LV-GFP-v5-TurboID) and A549 LV-EPN1-v5-TurboID cells were evaluated via confocal microscopy. The images show nuclei (DAPI, blue) and v5 expression (v5, green). **D)** A549 LV-EPN1-v5-TurboID and LV-GFP-v5-TurboID cells were mock- or IAV-infected (A/WSN/33) at MOI 100 for 30 minutes on ice and then incubated at 37°C for 30 minutes in the presence of 500µM biotin. Following cell lysis, biotinylated proteins were enriched for with streptavidin beads and all fractions evaluated via western blot for overall biotinylation (streptavidin), construct expression (v5), protein levels (β-actin) and presence of EGFR. **E)** A549 LV-EPN1-v5-TurboID cells were treated with DMSO (0.2%), NH_4_Cl (25mM) or Dynasore (80µM) for 30 minutes and then inoculated with A/WSN/33 at an MOI of 10 in their presence. At 4.5 hours post-infection, cells were fixed, permeabilized and stained for IAV nucleoprotein (NP, green) and DAPI (blue). Images were acquired with a DMIL fluorescent microscope. **F)** Relative Fluorescence Units (RFU) from **E**. Following subtraction of background fluorescence, values were normalized to those obtained upon DMSO treatment. Dashed line indicates RFU from uninfected cells. Statistical significance was inferred by two-tailed, one sample t-test against a theoretical mean of 100. * p < 0.05, ** p < 0.01. **G)** A549 LV-EPN1-v5-TurboID cells were treated as in **E** for 1.5 hours before cell viability was determined. Values are shown relative to the DMSO control, which was set to 100%. Dashed line indicates 80% viability. **H)** A549 LV-EPN1- and LV-GFP-v5-Turbo ID cells were mock or IAV infected and biotinylated proteins were enriched as in **D**. The eluate fraction of three independent experiments was assessed through LC-MS/MS and analyzed via label-free quantification. Peptide intensities were processed to calculate the probability of specific bait-prey interactions (SAINT Probability score) and the log2 fold change (log2 FC) in peak intensity over the GFP-v5-TurboID mock-infected samples. Candidates with a SAINT Probability score ≥0.6 and log2 FC > 1 (dashed lines) were considered potential interactors. **I)** Venn diagram depicting potential epsin 1 interactors identified in **H** for LV-EPN1- or GFP-v5-TurboID cells under mock or IAV infection. The number of interactors identified in each category is denoted. **B-E)** Images are representative of N = 3 independent experiments. Scale bar is 10µm (**B**, **C**) or 25µm (**E**). **F, G)** Data are means ± standard deviation from N = 3 independent experiments.

To identify IAV internalization receptor candidates, we acquired the epsin 1 interactome during virus infection through proximity labelling given the described role of epsin 1 in IAV internalization [19]. Our approach consisted of generating an A549 cell line that stably overexpressed epsin 1 tagged with the biotin ligase TurboID [28] and inoculating it with IAV strain A/WSN/33 (H1N1) at a multiplicity of infection (MOI) of 100. Following synchronization of infection on ice, cells were incubated at 37ºC in medium supplemented with biotin, allowing for concurrent biotinylation and virus internalization. Biotinylated proteins were enriched with streptavidin beads and analyzed through mass spectrometry (Fig 1A). To validate construct expression, A549 cells that stably overexpress epsin 1 tagged with a v5 linker and TurboID biotin ligase (LV-EPN1-v5-TurboID) or an untagged counterpart (LV-EPN) were analyzed through immunofluorescence microscopy (Fig 1B). Given that both constructs are comparably localized in the cytoplasm, we concluded that the addition of C-terminal TurboID did not lead to an aberrant protein localization. As a control, we generated A549 cells that stably overexpress green fluorescent protein (GFP) tagged with v5-TurboID (LV-GFP-v5-TurboID) and compared its localization to that of EPN1-v5-TurboID (Fig 1C). As overexpressed GFP-v5-TurboID is localized in both the cytoplasm and nucleus, it encompasses the localization of EPN-v5-TurboID and is suitable to control for non-specific interactors. We next assessed the ability of EPN1-v5-TurboID to biotinylate EGFR, a known epsin 1 interactor and putative IAV internalization receptor [16,26]. To do so, A549 LV-EPN1- and LV-GFP-v5-TurboID cells were mock or IAV-infected and, after virus binding on ice, incubated at 37ºC for 30 minutes in the presence of biotin. Following enrichment of biotinylated proteins, the lysate and eluate fractions were assessed through immunoblotting (Fig 1D). While both baits led to comparable enrichment and overall biotinylation, EGFR was biotinylated more strongly by EPN1-v5-TurboID. Lastly, we aimed to confirm that IAV also enters in a dynamin-dependent manner in our system as epsin 1 has a described role in dynamin-dependent endocytosis [19]. Indeed, pre-treatment of cells with the dynamin inhibitor dynasore did not impair cell viability (Fig 1G) but led to a significant reduction in IAV nucleoprotein (NP) signal at 4 hours post-infection, indicating that virus entry is dynamin-dependent in this context (Fig 1E and 1F). Treatment with ammonium chloride (NH_4_Cl), which blocks endosomal acidification, was included as positive control. Dynasore pre-treatment significantly reduced replication of vesicular stomatitis virus encoding GFP (VSV-GFP) but not parainfluenzavirus 5 encoding GFP (PIV5-GFP), which enter host cells via CME or fusion at the cell surface, respectively, corroborating inhibitor specificity [30,31] (S1 Fig). Following the characterization of our system, A549 LV-EPN1- and LV-GFP-v5-TurboID cells were inoculated with A/WSN/33 at MOI 100 in the presence of biotin and the enriched biotinylated protein fraction analyzed via mass spectrometry. After analysis of peptide signal intensities through label-free quantification, bait-prey interactions were scored with SAINTexpress and the fold change in protein abundance over the control condition, uninfected A549 LV GFP-v5-TurboID cells, was calculated (Fig 1H and S1 Table) [32]. Proteins were considered potential interactors if their fold change in abundance was greater than 2 and their SAINT probability score was higher than or equal to 0.6 [33]. These criteria yielded 1 potential GFP interactor, which was discarded from further studies, and 42 potential epsin 1 interactors: 8 specific to mock-infection, 13 to IAV-infection and 21 shared across both conditions (Fig 1I and S1 Table).

### Neo1 is a potential epsin 1 interactor and IAV receptor candidate

**Fig 2:**
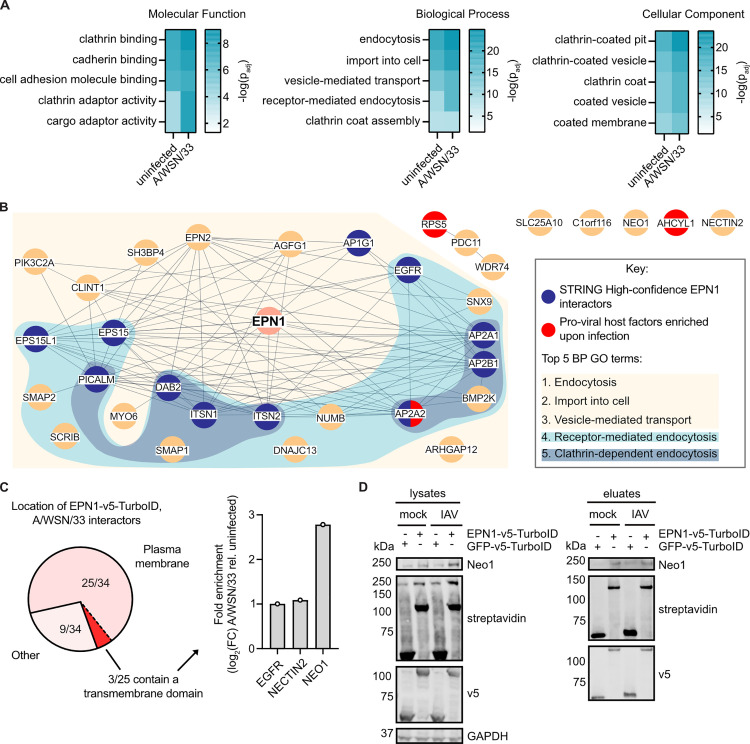
Identification of Neo1 as an IAV receptor candidate. **A)** Potential epsin 1 interactors detected under mock- or IAV-infection were assessed for enrichment of GO terms related to Molecular Function, Biological Process and Cellular Component with g:Profiler. Significance was determined with a Benjamini-Hochberg FDR threshold of 0.05. The five most enriched terms within each category for the mock-infected sample and the corresponding values in the infected sample are shown. Terms are depicted in descending order according to their –log (p_adj_) values. **B)** A STRING network of potential epsin 1 interactors detected in the IAV-infected sample was generated using StringApp in Cytoscape. High-confidence functional and physical protein associations are depicted (score >0.7). Nodes colored in blue or red are high-confidence interactors of epsin 1 or host factors enriched upon infection deemed as proviral, respectively [34]. The five most enriched Biological Process categories and the proteins involved in them are shown. **C)** The localization of potential interactors detected under IAV infection was subdivided into plasma membrane (GO:0005886) and all others according to the Cellular Component analysis in **A**. Plasma membrane proteins were evaluated for the presence of transmembrane domains using DeepTMHMM 1.0 [35] and their fold enrichment upon IAV infection relative to the uninfected condition was calculated. **D)** HEK293T cells were co-transfected with plasmids encoding for Neo1 and EPN1- or GFP-v5-TurboID. At 48 hours post-transfection, cells were mock- or IAV-infected at an MOI of 50 in the presence of biotin. Following enrichment of biotinylated proteins, the lysate and eluate fraction were evaluated via western blot for biotinylation (streptavidin), construct expression (v5), protein levels (GAPDH) and the presence of Neo1.

To characterize the epsin 1 interactome, potential interactors were initially evaluated by gene ontology (GO) term overrepresentation analysis (Fig 2A and S2 Table). Potential interactors detected in uninfected conditions were enriched for molecular function GO terms such as adaptor activity and binding of clathrin, cadherin or cellular adhesion molecule, for biological processes that describe endocytosis or import into cells and for cellular components that include clathrin-coated vesicles or pits. In sum, multiple terms that are directly annotated to or are associated with wildtype epsin 1 function and location were over-represented [19,36]. With the exception of clathrin-coat assembly, which was replaced by clathrin-mediated endocytosis, the terms in Fig 2A were also the top 5 most enriched terms under virus infection. To further assess the interactome, we generated a network depicting high-confidence functional and physical associations between interactors detected in IAV-infected conditions with STRING (Fig 2B). Therein we detected 3 proviral host factors enriched upon IAV infection [34] and 11 high-confidence epsin 1 interactors, including EGFR [37], providing strong validation for our interaction screen.

In order to identify receptor candidates, potential interactors detected under virus infection described by a ‘plasma membrane’ GO term annotation were screened for transmembrane domains with DeepTMHMM 1.0 (Fig 2C) [35]. This analysis yielded three receptor candidates: EGFR, Nectin2 and Neo1. To calculate their enrichment upon infection, their log2 fold change in abundance under IAV-infected or uninfected conditions over the control sample was compared. While EGFR and Nectin2 were not enriched, Neo1 was enriched 2.78-fold upon virus infection. To exclude enrichment biased by a high abundance at the cell surface, the A549 cell surface proteome published by [38] was analyzed (S2A Fig). While EGFR and Nectin2 were the 11^th^ and 25^th^ most abundant proteins, Neo1 was ranked 125^th^ out of 428 possible surface proteins, indicating a potentially specific enrichment upon IAV infection. Moreover, we corroborated that Neo1, akin to EGFR and Nectin2, is expressed in primary bronchial epithelial cells across all cell clusters (S2B Fig) [39]. Notably, the recently described putative IAV internalization receptor mGluR2 [12] remained undetected in the primary human airway cultures, highlighting that additional internalization receptors are yet to be identified. To confirm that Neo1 is a potential epsin 1 interactor, HEK293T cells were transiently transfected to express epsin 1- or GFP-v5-TurboID in conjunction with Neo1 and infected with A/WSN/33 at MOI 50 in the presence of biotin. Following streptavidin-bead enrichment, cell lysates and eluate fractions were evaluated via immunoblotting (Fig 2D). Upon co-expression with EPN1-v5-TurboID, the biotinylated form of Neo1 could be readily detected. Overall, these data show that Neo1 is a potential epsin 1 interactor and IAV receptor candidate.

### Neo1 specifically interacts with IAV

**Fig 3:**
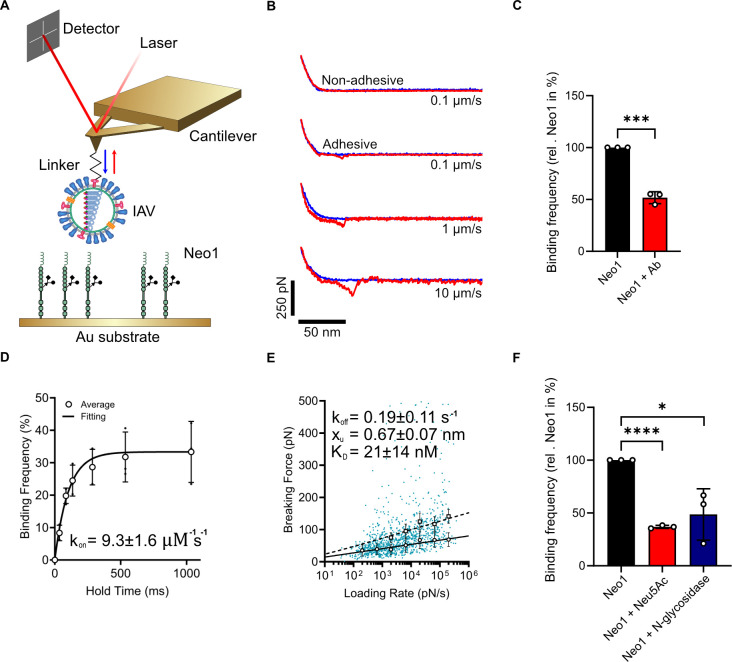
Assessment of IAV binding to Neo1 using Atomic Force Microscopy. Thermodynamic and dynamic characterization of IAV-Neo1 interactions on model surfaces. **A)** Schematic representation of the experimental setup for quantifying IAV-Neo1 interactions. AFM cantilevers functionalized with IAV were approached to a Neo1-coated model surface. Virus schematic adapted from [29]. **B)** During approach (blue curve), the virus is brought into contact with the Neo-1 surface. Upon retraction, some adhesive curves are recorded in which adhesion events are observed. Adhesion force increases with the retraction velocity. **C)** Binding probability of IAV-Neo1 interactions was measured in the presence of anti-Neo1, confirming interaction specificity. **D)** Binding probability between IAV-functionalized tips and a Neo1-coated surface was measured at varying contact times. An exponential decay model was fitted to calculate *k*_*on*_. **E)** Dynamic force spectroscopy plot of the rupture forces across eight orders of magnitude of loading rates. Data were fitted using the Bell-Evans model (solid line) for single interactions and the Williams-Evans model (dotted lines) used to predict double interactions. **F)** Binding probability was measured after treating Neo1-coated surfaces with Neu5Ac or N-glycosidase, highlighting the role of sialic acid and glycosylation in the interaction. **C, D, E, F)** Data represent mean ± standard deviation (SD) from N = 3 independent experiments. Statistical significance was inferred by two-tailed unpaired t-test (**C**, **F**). *p < 0.05, ***p < 0.001, ****p < 0.0001.

To further investigate the role of Neo1 as a receptor for IAV, we conducted atomic force microscopy (AFM)-based single-molecule force spectroscopy (SMFS) experiments to assess the binding properties of individual IAV virions to Neo1 (Fig 3A). To this purpose, IAV virions were covalently attached to AFM tips via a flexible PEG_24_ linker using established protocols [40,41]. Neo1 receptors were immobilized on a gold surface using NHS-EDC chemistry, ensuring stable and specific grafting. Force-distance (FD) curves were recorded through consecutive approach-retraction cycles of the AFM tip to the surface, capturing the formation and subsequent rupture of the IAV-Neo1 complex (Fig 3A and 3B). Binding frequency (BF) was determined from FD curves showing adhesive events between the IAV-functionalized tip and the Neo1-coated surface, calculated as the ratio of specific events to total curves. Specificity was validated by adding a Neo1 antibody, which reduced BF (Fig 3C).

To investigate binding kinetics, we examined the dependence of BF on contact time (Fig 3D) and observed an exponential decay [42]. From these data, we calculated a high association rate (*k*_*on*_=9.3 ± 1.6 µM^-1^ s^-1^) between IAV and Neo1. The dissociation constant was measured using dynamic force spectroscopy (DFS). FD curves were recorded at various loading rates (LR), derived from different tip retraction velocities (0.1–10 µm/s). The DFS plot (Fig 3E) revealed a linear relationship between LR and breaking force, characteristic of virus-receptor interactions [43]. The data were analyzed using the Bell-Evans model, which hypothesizes a single energy barrier in the binding free-energy landscape between the bound and unbound states. This analysis yielded a dissociation rate constant (k_off_=0.19 ± 0.11 s^-1^) and distance to the transition state (x_u_ = 0.64 ± 0.07 nm). From these, the dissociation constant (K_D_ = 21 ± 14 nM) was calculated, indicating a high-affinity interaction between IAV and Neo1. Additionally, Williams-Evans predictions for double bond forces (dotted line, Fig 3E) aligned well with observed double-interaction rupture forces, suggesting the capacity of individual virions to establish multiple bonds with Neo1 receptors.

To explore the role of sialic acid and glycosylation in the binding process, we measured the changes in binding probability after treating Neo1 with Neu5Ac and N-glycosidase. Incubation of IAV with 1mM Neu5Ac (Fig 3F) decreased binding probability with Neo1 by 37 ± 1%, indicating competition between sialic acid and Neo1 for IAV binding (Fig 3F). Similarly, deglycosylation of Neo1 using N-glycosidase [44] decreased binding probability by 40 ± 20%, suggesting a preference for N-linked glycans. These findings suggest that IAV primarily binds to sialic acid on N-glycans in Neo1.

### Neo1 is a proviral host factor in IAV infection

**Fig 4:**
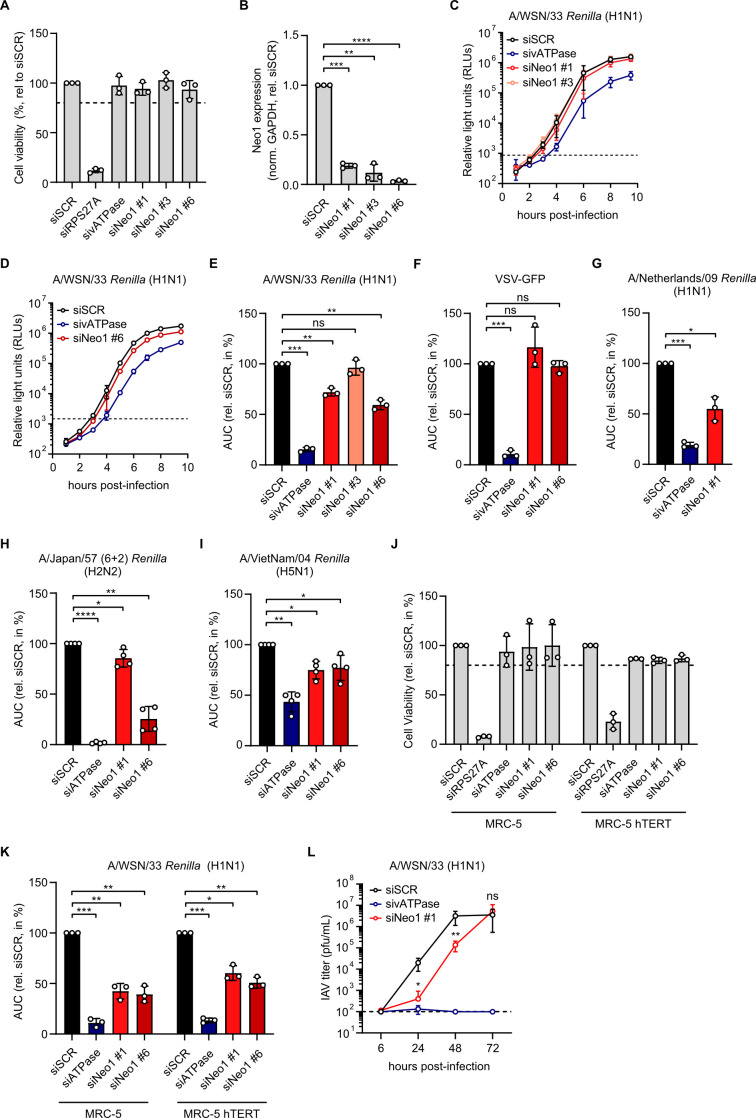
Neo1 plays a proviral role in IAV infection. **A)** A549 cells were treated with 30nM of control (scrambled, SCR), RPS27A-, vATPase- or Neo1 (#1, #3 or #6)- targeting siRNAs for 48 hours. At this time, cell viability was assessed. **B)** A549 cells were treated as in **A** and Neo1 knockdown efficacy was determined via RT-qPCR. Changes in Neo1 expression were determined with the 2^–∆∆Ct^ method [47] using GAPDH as an internal control. **C, D)** A549 cells were treated as in **A** using siRNAs against Neo1 #1, 3 in **C** and #6 in **D** and inoculated with A/WSN/33 *Renilla* at an MOI of 1. Following a 1 hour adsorption, luminescence was measured at the indicated hours post-infection. Dashed line indicates background luminescence from mock-infected cells. **E)** The area under the curve (AUC) was calculated for **C** and **D** until 8 hours post-infection. **F)** A549 cells treated as in **A** using siRNAs against Neo1 #1, 6 were inoculated with VSV-GFP at an MOI of 3. The GFP signal was measured at the indicated times post-infection and the total integrated green signal intensity was normalized to cell confluence. AUC values were calculated until 9 hours post-infection. **G-I)** A549 cells were treated with siSCR, sivATPase or siNeo1 for 48 hours and inoculated with A/Netherlands/602/09 *Renilla* at an MOI of 3 (**G**), H2-pseudotyped *Renilla* with internal and *Renilla* segments from A/WSN/33 *Renilla* as well as A/Japan/57 N2 generated on H2-expressing cells at an MOI of 3.5 (**H**) or A/VietNam/1203/04 *Renilla* at an MOI of 1 (**I**). Luminescence was monitored as in **C**. AUC values were calculated until 9.5 (**G**), 9 (**H**) or 7 (**I**) hours post-infection. **J, K)** MRC-5 and MRC-5 hTERT were treated with siRNAs as in **A**. At 48 hours post-transfection, cell viability was assessed (**J**) or cells were inoculated with A/WSN/33 *Renilla* at an MOI of 1 and luminescence measured as in **C**. AUC values were calculated until 8 hours post-infection. **L)** siRNA-treated A549 cells were inoculated with A/WSN/33 at an MOI of 0.01 and supernatant was collected at 6, 24, 48 and 72 hours post-infection. Viral titers were determined via plaque assay. **A-L)** Data are means ± standard deviation from N = 3 (**A-G**, **J-L**) or N = 4 (**H, I**) independent experiments. **B, E-I, K-L)** Statistical significance was inferred by unpaired two-tailed t-test with Welch’s correction on the log-transformed data (**L**) or two-tailed one sample t-test with a theoretical mean of 1.0 (**B**) or 100 (**E-I**, **K**). *p < 0.05, **p < 0.01, ***p < 0.001, ****p < 0.0001, ns = not significant. **A, J)** Dashed line indicates 80% viability.

The role of Neo1 in IAV infection is thus far unexplored. To address this, A549 cells were treated with individual Neo1-targeting siRNAs and their effect on A/WSN/33 *Renilla* reporter virus [45] replication evaluated. An siRNA targeting cellular vacuolar ATPase, a factor required for endosomal acidification and IAV entry [46], and a non-targeting counterpart (siSCR) were included as positive and negative controls, respectively. Importantly, treatment with the siRNAs did not impair cell viability (Fig 4A) and was effective in decreasing Neo1 expression (Fig 4B). Moreover, treatment with siNeo1 #1 or #6 led to significantly reduced A/WSN/33 *Renilla* virus replication, yielding area under the curve (AUC) values of 72 and 59%, respectively, relative to the siSCR control (Fig 4C–4E). Given that pre-treatment with these siRNAs did not lead to a decrease in replication of VSV-GFP (Fig 4F), which also enters cells by endocytosis, we concluded their effect to be specific to IAV. Next, the effect of Neo1 knockdown on replication of three additional *Renilla* reporter viruses from subtypes H1 (Fig 4G), H2 (Fig 4H) and H5 (Fig 4I) was assessed. For all three viruses knockdown of Neo1 expression decreased virus replication significantly, indicating that the effect is not strain- or subtype-specific. In addition, depletion of Neo1 expression in unmodified and hTERT-immortalized MRC-5 cells impaired A/WSN/33 *Renilla* virus replication without conferring cytotoxicity, highlighting that the effect is shared in immortalized and primary cells alike (Fig 4J and 4K). Finally, we investigated the role of Neo1 on multicycle growth of wildtype A/WSN/33 in A549 cells. Treatment with siNeo1 #1 resulted in reduced viral titers at 24 and 48 hours post-infection compared to the siSCR control (Fig 4L). These data support the findings with the *Renilla* reporter virus and suggest that diminished Neo1 expression can lead to a disadvantage in viral growth. Overall, we conclude that Neo1 plays a proviral role in infection of IAVs of multiple subtypes.

### Neo1 can co-localize with incoming IAV and affects virus entry

**Fig 5:**
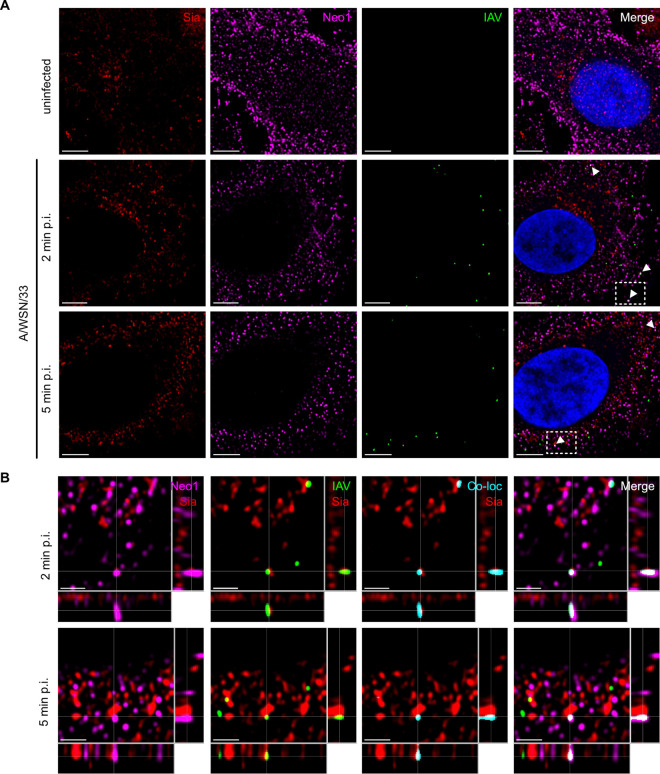
Neo1 can co-localize with incoming virus at the cell surface. **A)** A549 LV-Neo1 cells were inoculated with A/WSN/33 at an MOI of 100 and incubated for 2 or 5 minutes-post infection at 37°C. Fixed, non-permeabilized samples were probed with WGA-AF488 lectin, anti-Neo1 and anti-IAV HA antibodies and their expression assessed through confocal microscopy, 63x objective. Images are Z-stack slices that depict sialic acid (Sia, red), Neo1 (magenta), IAV HA (green) and cell nuclei (DAPI, blue). White arrows indicate Neo1 and IAV co-localization. Scale bar is 5µm. **B)** Orthogonal section of A549 LV-Neo1 cells inoculated with A/WSN/33 at 2 or 5 minutes post-infection depicted in **A** (enclosed by a dashed white box). An intensity-based co-localization channel (cyan) is included to facilitate viewing of Neo1 and IAV HA co-localization. Images are representative of N = 2 independent experiments. Scale bar is 2µm.

Cellular receptors interact with incoming virus at the cell surface, likely triggering a signalling cascade that results in virus uptake and may lead to co-internalization. To further assess the potential of Neo1 as an IAV receptor, we infected A549 cells stably overexpressing Neo1 (LV-Neo1) with A/WSN/33 at MOI 100 and evaluated their localization at 2 and 5 minutes post-infection under non-permeabilized conditions via immunofluorescence (Figs 5 and S4). Cell surface sialic acid, detected with Alexa Fluor 488-labelled WGA lectin, was included as a marker. Instances of IAV co-localization with Neo1 in cells where Neo1 expression is non-saturating, i.e., where it remains distinguishable and dot-like, were readily detectable and corroborated with orthogonal sectioning (Figs 5A, S4A, 5B and S4B, respectively). However, we also detected numerous IAVs that did not co-localize with Neo1 (Figs 5A and S4A). This indicates that while incoming IAV can co-localize with cell surface Neo1, as expected for a potential receptor, a stringent co-localization is not required at early stages of viral entry.

**Fig 6:**
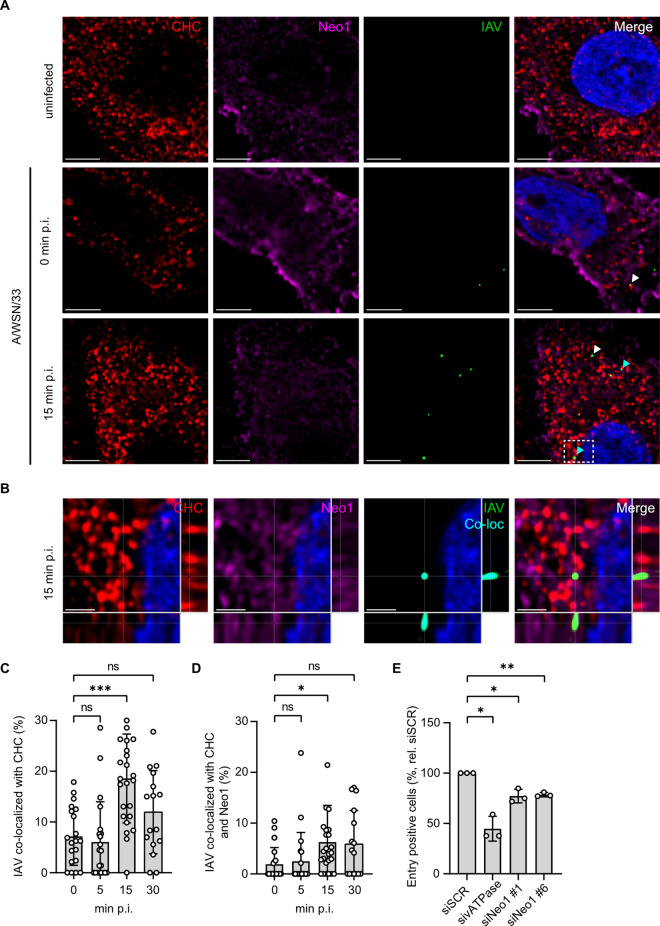
Analysis of Neo1 co-localization with incoming virus and clathrin at early times post-infection and its role on virus entry. **A)** A549 LV-Neo1 cells were inoculated with A/WSN/33 at an MOI of 25 and, following synchronization of infection on ice, incubated for 0, 5, 15 or 30 minutes at 37°C. Fixed, permeabilized samples were probed with anti-clathrin heavy chain (CHC)-AF488, anti-Neo1 and anti-IAV HA antibodies and their expression assessed through confocal microscopy, 63x objective. Images are Z-stack slices from uninfected cells and those fixed at 0 or 15 minutes post-infection that depict CHC (red), Neo1 (magenta), IAV HA (green) and cell nuclei (DAPI, blue). White and cyan arrows indicate co-localization between CHC and IAV alone or in addition to Neo1, respectively. **B)** Orthogonal section of A549 LV-Neo1 cells inoculated with A/WSN/33 maintained for 15 minutes post-infection shown in **A** (enclosed by dashed white box). An intensity-based co-localization channel (cyan) is included to facilitate viewing of Neo1 and IAV HA co-localization. **C)** Deconvolved images from **A** were analyzed with Imaris to identify cells, IAV and clathrin spots. IAV at a distance less than 0.35µm to CHC were deemed to co-localize. The percentage of CHC-IAV co-localizing spots over total IAV spots per cell is shown. **D)** Images from **A** were analyzed as in **C**. Using an intensity-based co-localization channel to identify instances of IAV and Neo1 co-localization, spots were rendered to count Neo1-co-localized IAVs. Triple co-localization was inferred when IAV was at a distance less than 0.35µm to CHC and co-localized IAV spots. The percentage of IAVs co-localizing with CHC and Neo1 over total IAVs per cell is shown. **E)** MRC-5 cells were treated with control, vATPase- or Neo1- targeting siRNAs for 48 hours and then infected with BlaM1 VLPs pseudotyped with the HA and NA from A/WSN/33. At 5 hours post-infection, entry positive cells were detected by flow cytometry. Data are means ± standard deviation of N = 3 independent experiments. Statistical significance was inferred by unpaired, two-tailed one sample t-test. *p < 0.05, p** < 0.01, ns = not significant. **A, B)** Images are representative of N = 3-4 independent experiments. Scale bars represent 4 and 2µm, respectively. **C, D)** Data are means ± standard deviation of N = 3-4 independent experiments, where at least three cells were analyzed per time point for each replicate. Each dot represents one cell. Statistical significance was inferred by unpaired, non-parametric One Way ANOVA with the Kruskal-Wallis multiple comparison test. *p < 0.05, ***p < 0.001, ns = not significant.

To investigate if Neo1 and IAV may co-internalize, we evaluated their localization at later stages of viral entry. To do so, A549 LV-Neo1 cells were inoculated with A/WSN/33 at MOI 25 and, following synchronization of viral infection on ice, incubated at 37ºC for 0, 5, 15 or 30 minutes prior to fixation. The localization of Neo1, IAV and clathrin in permeabilized samples was assessed through immunofluorescence (Fig 6A). IAV and clathrin co-localized in a time-sensitive manner (white arrows) and instances of triple co-localization were also detected (cyan arrows; Fig 6A and 6B). Their co-localization was quantified through object- and intensity-based co-localization. IAV and clathrin were rendered as spot objects and incorporated into segmented cells. As object-based rendering did not capture the Neo1 signal accurately, an intensity-based co-localization channel between Neo1 and IAV was generated and its signal rendered into spots to count instances of IAV and Neo1 co-localization. Thus, the percentage of IAVs co-localizing with clathrin alone or in conjunction with Neo1 per cell was obtained (Fig 6C and 6D). We observed that the peak of IAV and clathrin co-localization occurs at 15 minutes post-infection (Fig 6C), which coincides with a less prominent peak describing IAV, clathrin and Neo1 co-localization (Fig 6D). Since VSV is internalized by CME but does not require Neo1 for entry (Fig 4F), we next evaluated the localization of Neo1, clathrin and VSV at early times post-infection to provide context (S5G and S5H Fig). Similar to IAV, a peak in VSV, clathrin and Neo1 co-localization was observed at the peak of VSV and clathrin co-localization. Half of the virus and clathrin co-localized spots were also positive for Neo1, rendering it difficult to discern the specificity of Neo1 co-localization in this setup. For both experiments, the Neo1 mean fluorescence intensity (MFI) per image, IAV or VSV and clathrin spots per cell was comparable across the time points tested (S5A–S5F Fig). To gauge the potential of Neo1 as an internalization receptor, we next assessed whether its absence impacted early virus entry via the BlaM1 virus-like-particle (VLP) assay (Fig 6E). Depletion of Neo1 expression in MRC-5 cells with siRNAs led to a statistically significant reduction in entry of BlaM1 VLPs pseudotyped with A/WSN/33 H1N1, indicating a proviral role for Neo1 in viral entry. Given that Neo1 can co-localize with IAV at early times post-infection and affect virus entry, this suggests that it may act as a potential IAV receptor.

## Discussion

Entry is an essential, early step in the viral lifecycle that is reliant on a multitude of host factors. Following cellular attachment through binding to sialic acid, IAVs are hypothesized to engage with a signalling receptor(s) that triggers viral uptake [9,13,16], yet the extent of the internalization receptor repertoire is unclear. Here, we performed proximity labelling centered on epsin 1 during infection and uncover Neo1 as a potential IAV internalization receptor. We showed that Neo1 is a proviral host factor that can interact with A/WSN/33 IAV through its N-linked glycosylations, co-localize with incoming virus at early times post-infection and affect viral entry.

Since entry of IAVs can occur via multiple endocytic routes, others have speculated that route-specific factors might not be identified through genome-wide RNAi approaches [4]. Thus, we performed TurboID-based proximity labelling to identify proteins neighboring epsin 1 during early stages of viral entry. Our epsin 1 interactome was over-represented for GO terms pertaining to wild type epsin 1 functions and localization and contained 11 known interactors. Contrary to our expectations, we did not find clathrin, although this could be explained by the limited ~35nm biotinylation radius of TurboID [48]. Moreover, RPS5, AHCYL1 and AP2A2, host factors with presumed proviral roles in IAV infection [34], were enriched upon infection in our system. Myo6 and EGFR, host factors involved in IAV entry [16,49], were detected but not enriched upon infection. This could be explained since the role of Myo6 in IAV uptake is dispensable in non-polarized environments, such as A549 cells [49]. Although EGFR’s role in promoting IAV uptake has been studied in A549 cells [16], it is not known whether its internalization upon viral infection is dependent on epsin 1. Since our system relies on epsin 1 overexpression, it is possible that sensitive interactions are considered non-enriched yet a potential role in infection should not be ruled out. In sum, we identify multiple proteins in the vicinity of epsin 1 during infection, providing a list of host factors potentially involved in CME of IAV.

From our analysis of the interactome hits for plasma membrane localization and infection-induced epsin 1 vicinity, Neo1 emerged as a promising candidate for an internalization receptor. Neo1 is a member of the immunoglobulin superfamily (IgSF) and acts as a cell surface receptor for netrin 1 [50], repulsive guidance molecules (RGMs) [51,52] and bone morphogenetic proteins (BMPs) [53]. Interestingly, binding of RGMa to Neo1 can activate Rho GTPases and trigger subsequent actin remodeling [54], which has been described to aid in IAV internalization [49,55]. Neo1 is ubiquitously expressed and has been implicated in neuronal development and axonal guidance in the central nervous system [52,56], iron hemostasis in hepatocytes [57] and cell migration and adhesion [58], amongst others. Importantly, we detected Neo1 expression in human bronchial epithelial cells, a primary site of IAV infection.

The characteristics of an internalization receptor include a proviral role in entry, the ability to interact with virions and co-localization with incoming virus at the cell surface and during uptake. Depletion of Neo1 expression in A549 cells through siRNAs led to a reduction in replication of two H1, an H2 and an H5 reporter virus within a linear scale. Importantly, this effect was conserved when performing the same experiment in primary MRC-5 cells. This reduction is observed at early times post-infection, consistent with a proviral factor involved in viral entry, and translated to diminished viral titers when testing multicycle growth of authentic virus. Since VSV replication remained unaffected, the role of Neo1 appears to be specific to CME of IAV rather than CME in general.

Akin to our data, the pharmacological inhibition, knockdown or knockout of host proteins involved in IAV internalization identified by others, such as EGFR or mGluR2, can impair but not abolish infection [12,16,18]. Although inhibition or depletion of expression could be incomplete, signalling receptor redundancy has been speculated before [16] and may indeed be the case. This is further complicated since IAVs can enter cells via multiple routes and knockdown of adaptor proteins essential to a given route [19] does not affect overall viral uptake. For example, in the absence of Neo1 expression, increased EGFR-dependent uptake could take place. Thus, we speculate that entry route redundancy is the reason for the modest decrease in viral replication levels upon knockdown of Neo1.

The diversity in IAVs underscores intrinsic differences in viral entry. Different IAV subtypes can lead to the activation of different signalling pathways [59] and virion morphology can impact the entry route [21,22,24], meaning that distinct receptors could be engaged. In support of this, not all IAVs bind to sialic acid, as exemplified by the bat IAVs of the H17 and H18 subtypes that use MHC-II for host cell entry [60–63]. Thus, the amount of receptor candidates identified so far could both reflect differences in IAV strains and cell lines used. Future research could study IAV entry factors in stratified primary human airway cultures in order to elucidate the internalization receptor repertoire therein and grasp differences between IAV subtypes.

After determining that Neo1 has a proviral function, we next assessed its ability to interact with IAV. Through AFM, we captured the formation and rupture of the IAV-Neo1 complex. We determined that Neo1 can specifically interact with IAV with high-affinity. Moreover, the binding process appears to be dependent on sialic acid in N-linked glycans on Neo1. However, treatment with an N-glycosidase did not completely abolish the binding, indicating that additional factors contribute to the interaction. Thus far, interactions between IAV HA to cell surface host factors have been described as partially dependent on N-linked glycosylation [13] or as glycosylation independent [12]. In addition, certain IAVs can attach to and enter cells independently of sialic acid, suggesting that proteinaceous receptors could facilitate IAV uptake [39]. This draws a parallel to Reoviruses, which attach to cells through binding to sialic acid and the proteinaceous receptor junctional adhesion molecule-A (JAM-A), prior to internalization via β1 integrin [64–66].

Next, we evaluated the co-localization of Neo1 and IAV at early stages of infection through confocal microscopy. We observed that surface Neo1 can co-localize with incoming IAV at 2 and 5 minutes post-infection. Although the co-localization is not complete, this has also been the case for other receptor candidates [16]. We also detected that Neo1 can co-localize with IAV and clathrin at times where their co-localization is highest. Notably, we found a lower percentage of IAVs co-localizing with clathrin than previously reported [19], but this could be explained by differences in IAV strains, cell lines and image analysis. As there are less instances of triple co-localization compared to just IAV and clathrin, Neo1 appears to be dispensable for CME of IAV. Since we observed a similar trend for VSV, we could not infer a specific co-internalization based on these data. Future work could address this through the use of live cell microscopy approaches in cells with higher endogenous Neo1 expression, which could additionally shed further light on the route of Neo1-mediated IAV entry and the role of other host proteins involved [67–69]. Notwithstanding, the depletion of Neo1 expression in MRC-5 cells impaired uptake of BlaM1 H1N1 VLPs, which simultaneously confirmed a proviral role in IAV uptake and highlighted the redundancy in entry discussed previously. Overall, the co-localization with incoming IAV and the proviral role in virus entry is consistent with what would be expected for an internalization receptor and thus, these data indicate that Neo1 could act as such.

In summary, we describe the acquisition of the epsin 1 interactome upon virus infection and uncover Neo1 as a novel proviral host factor for IAV. As Neo1 is expressed at the primary site of infection, can interact with IAV and co-localize with the virus at early times post-infection and its depletion impairs IAV entry, this study highlights its potential as one of the IAV internalization receptors.

## Materials and methods

### Cells

A549, HEK293T, MRC-5 and MDCK cells were acquired from ATCC (cat #CCL-185, #CRL-11268, #CCL-171 and #CCL-34, respectively). MRC-5 hTERT were described before [70]. A549, HEK293T and MDCK cell lines were cultured in Dulbecco’s Modified Eagle Medium (DMEM, Gibco) supplemented with 10% fetal calf serum (FCS, Gibco) and 1% penicillin-streptomycin (P/S, Gibco). MRC-5 and MRC-5 hTERT cells were cultured in Minimum Essential Medium Eagle (Sigma-Aldrich) supplemented with 10% FCS, 1% P/S, 2mM GlutaMAX (ThermoFisher Scientific) and 1% non-essential amino acids (ThermoFisher Scientific). All cell lines were maintained at 37°C and 5% CO_2_.

### Viruses

A/WSN/33, A/Netherlands/602/2009 and A/VietNam/1203/04 *Renilla* viruses were propagated and titrated as described [45]. The H2N2 reporter virus harboring the internal and *Renilla* segments from A/WSN/33 *Renilla* and N2 from A/Japan/305/1957 was generated as in [45] by using H2-expressing cells, whereby H2 was derived from strain A/Japan/305/1957. VSV, VSV-GFP and PIV5-GFP were kindly provided by Ben Hale, Institute of Medical Virology, University of Zurich.

### Proximity labelling and enrichment of biotinylated proteins

Proximity labelling was performed as done previously [28,70]. In brief, A549 cells stably expressing Epsin 1-v5-TurboID or GFP-v5-TurboID were seeded onto 6 well plates or 10 cm dishes for immunoblotting or proteomic analysis, respectively. Cells were grown overnight, starved in DMEM without FCS for 18–20 hours and then inoculated with A/WSN/33 at MOI 100 in DMEM for 30 minutes on ice. At this time, 500µM biotin (Sigma-Aldrich, catalog# B4501-1G) was added and the cells were incubated for 30 minutes at 37°C. After 5 washes with ice-cold PBS, cells were lysed by incubation in RIPA buffer (50 mM Tris-HCl pH 8, 150 mM NaCl, 0.1% SDS, 0.5% sodium deoxycholate, 1% Triton X-100) supplemented with PhosSTOP (Sigma-Aldrich, catalog# 4906845001) and cOmplete, Mini, EDTA-free Protease-Inhibitor-Cocktail (Sigma-Aldrich, catalog# 11836170001) at 4°C. Sonicated lysates were clarified by centrifugation at 10,000g at 4°C for 15 minutes and a fraction of the supernatant was diluted with Laemmli buffer (Bio-Rad, catalog# 1610747) to obtain input lysate samples. The remaining supernatant was incubated overnight with Pierce Streptavidin Magnetic Beads (ThermoFisher Scientific, catalog# 88817) at 4°C. Subsequently, the beads were washed in the following order: twice with RIPA lysis buffer, once with 1M KCl, once with Na_2_CO_3_, once with 1M urea in 10mM Tris-HCl (pH 8.0) and finally either twice with RIPA lysis buffer or three times with ABC buffer (50mM ammonium bicarbonate buffer, pH 8) for immunoblotting or proteomic analysis, respectively. For the former, proteins were eluted from beads by incubation with Laemmli buffer for 5 minutes at 95°C. For the latter, on-bead digestion was performed prior to mass spectrometry.

Proximity labelling in transfected HEK293T cells was performed similarly to above. To do so, co-transfected HEK293T cells were inoculated with A/WSN/33 at MOI 50 in DMEM for 30 minutes on ice and, following addition of biotin to 500µM, incubated for 30 minutes at 37°C. Following acquisition of cell lysates as per above, clarified lysates were incubated with Pierce Streptavidin Magnetic Beads for 1 hour at RT or overnight at 4°C to enrich for biotinylated proteins. Subsequently, the beads were washed and proteins eluted for immunoblotting analysis as described previously.

### Immunoblotting

Samples diluted in 1xLaemmli buffer were incubated at 95°C for 5 minutes and loaded onto Bolt 4–12% Bis-Tris Plus Mini Protein Gels (ThermoFisher Scientific, catalog# NW04125). Proteins were separated by SDS-polyacrylamide gel electrophoresis (SDS-PAGE) and transferred onto 0.45µm nitrocellulose membranes. To assess overall biotinylation, membranes were probed with 1:2500 IRDye 800CW Streptavidin (LI-COR Biosciences, catalog #926–32230) in PBS supplemented with 0.05% Tween-20 (PBS-T) and 0.05% SDS for 1 hour. After blocking with 5% milk in PBS-T for 1 hour, membranes were probed with primary antibodies in PBS-T overnight at 4°C, washed twice with PBS-T and incubated with secondary antibodies diluted 1:5000 in PBS-T for another hour. Following two washes with PBS-T, signal was detected using an Odyssey Fc imager (LI-COR Biosciences) and analyzed with Image Studio Light v.5.2 (LI-COR Biosciences). Signal from HRP-conjugated secondary antibodies was detected using SuperSignal West Dura Extended Duration Substrate (ThermoFisher Scientific, catalog #34075) and the Odyssey FC imager. The following primary antibodies were used: 1:1000 rabbit anti-Neo1 (Abcam, catalog #ab183511), 1:1000 mouse anti-GAPDH (Santa Cruz, catalog# sc-47724), 1:2000 mouse anti-v5 (Bio-Rad, catalog #MCA1360), 1:1000 anti-β-actin (Santa Cruz, catalog# sc-47778) and 1:1000 anti-EGFR (Cell Signalling, catalog# 4267). The following secondary antibodies were used at a 1:5000 dilution: IRDye 680RD Goat anti-Mouse IgG (H + L) (LI-COR Biosciences, catalog# 926–68070), IRDye 800CW Goat anti-Mouse IgG (H + L) (LI-COR Biosciences, catalog# 926–32210), IRDye 680RD Goat anti-Rabbit IgG (H + L) (LI-COR Biosciences, catalog# 926–68071), IRDye 800CW Goat anti-Rabbit IgG (H + L) (LI-COR Biosciences, catalog# 926–32211) and Anti-Rabbit IgG (whole molecule)–Peroxidase (Sigma-Aldrich, catalog# A0545).

### LC-MS/MS and data analysis

Mass spectrometry was performed in collaboration with the Functional Genomics Center Zurich (FGCZ) at the University of Zurich. For each sample, the washed beads were re-suspended in 50 µl digestion buffer (triethylammonium bicarbonate (TEAB), pH 8.2). Proteins were on-bead digested using 5 µl of Sequencing Grade Trypsin (100 ng/µl in 10 mM HCl, Promega). The digestion was carried out in a microwave instrument (Discover System, CEM) for 30 min at 5 W and 60 °C. The supernatants were transferred in new tubes and the beads were washed with 150 µl trifluoroacetic acid (TFA) -buffer (0.1% TFA, 50% acetonitrile) and combined with the first supernatant. The samples were dried to completeness and re-solubilized in 20µL of MS sample buffer (3% acetonitrile, 0.1% formic acid).

LC-MS/MS analysis was performed on an Q Exactive mass spectrometer (ThermoFisher Scientific) equipped with a Digital PicoView source (New Objective) and coupled to a nanoAcquity UPLC (Waters Inc.). Solvent composition at the two channels was 0.1% formic acid for channel A and 0.1% formic acid, 99.9% acetonitrile for channel B. Column temperature was 50°C. For each sample 4 μL of peptides were loaded on a commercial Symmetry C18 trap column (5 µm, 180 µm x 20 mm, Waters Inc.) connected to a BEH300 C18 column (1.7 µm, 75 µm x 150 m, Waters Inc.). The peptides were eluted at a flow rate of 300 nL/min with a gradient from 5 to 35% B in 60 min, 35–60% B in 5 min and 60–80% B in 10 min before equilibrating back to 1% B.

The mass spectrometer was operated in data-dependent mode (DDA) using Xcalibur at a heated capillary temperature at 275 °C. Full-scan MS spectra (350 − 1500 m/z) were acquired at a resolution of 70’000 at 200 m/z after accumulation to a target value of 3’000’000, followed by HCD (higher-energy collision dissociation) fragmentation on the twelve most intense signals per cycle. Ions were isolated with a 1.2 m/z isolation window and fragmented by higher-energy collisional dissociation (HCD) using a normalized collision energy of 25%. HCD spectra were acquired at a resolution of 35’000 and a maximum injection time of 120 ms. The automatic gain control (AGC) was set to 100’000 ions. Charge state screening was enabled and singly and unassigned charge states were rejected. Only precursors with intensity above 25’000 were selected for MS/MS. Precursor masses previously selected for MS/MS measurement were excluded from further selection for 40 s.

The mass spectrometry proteomics data were handled using the local laboratory information management system (LIMS) [71] and all relevant data have been deposited to the ProteomeXchange Consortium via the PRIDE (http://www.ebi.ac.uk/pride) [72] partner repository with the data set identifier PXD058908.

The mass spectrometry data was analyzed by label-free quantification using FragPipe v.16 and the MSFragger Search Engine [73], as done in [70]. Spectra were searched against the human reference proteome (UP000005640) and IAV A/WSN/33 proteomes (UP000000834). As in [70], trypsin protein digestion with an allowance for 2 missed cleavages, a precursor mass tolerance of 50PPM and a fragment mass tolerance of 20PPM were set. Methionine oxidation and N-terminal acetylation were selected as variable modifications. The peptide intensities from three independent replicates were analyzed with SAINTexpress under default settings ([32]; lowMode = 0, minFold = 1, normalize = 1). Uninfected samples from A549 LV-GFP-v5-TurboID cells were utilized as negative controls. To determine potential interactors, the following criteria were utilized: Saint Probability score ≥ 0.6 [33] and fold change A (FCA) in abundance relative to the negative control > 2.

### Cloning

To generate pLVX-EPN1-v5-TurboID and pLVX-EPN1, epsin 1 was amplified from pEGFP-C1-EPSIN 1 (Addgene plasmid no. 22228 from [74]*,* a kind gift from Pietro De Camilli) and cloned into pLVX-v5-TurboID or pLVX-IRES-puro plasmid using either NotI and EcoRI or EcoRI and XhoI restriction enzymes, respectively. To generate pLVX-Neo1, Neo1 was amplified from pCMV6-Neo1, which encodes the cDNA of human Neo1 transcript variant 1 (Origene, catalog# SC118624), and cloned into pLVX-IRES-puro plasmid with XhoI and XbaI restriction enzymes. The pLVX-v5-TurboID, pLVX-GFP-v5-TurboID and pLVX plasmids were a kind gift from Ben Hale, Institute of Medical Virology, University of Zurich [70]. The primers utilized are listed in S3 Table. All inserts were verified by sequencing.

### Generation of lentiviruses and cell lines

Lentiviruses were generated by co-transfecting HEK293T cells with 1μg of pCMV-DR8.91, 1μg of pMD2.G and 1.7μg of pLVX-IRES-puro plasmid encoding a desired construct (Neo1, Epsin 1, Epsin 1-v5-TurboID, GFP-v5-TurboID) using FuGene (Promega, catalog# E2311) as per manufacturer’s recommendations. At 48 and 72 hours post-transfection, supernatants were collected, clarified by centrifugation at 1500rpm for 5 minutes and filtered through 0.45μm membrane filters prior to use.

To generate cell lines stably expressing a given construct, A549 cells were transduced with a corresponding lentivirus in the presence of 8µg/mL polybrene (Sigma-Aldrich). At 48 hours post-transduction, cells were subjected to 1µg/mL puromycin selection (ThermoFisher Scientific, catalog# A1113803).

### Inhibitor treatment

A549 LV-EPN1-v5-TurboID cells were seeded at 12,000 cells per well into a 96 well plate and grown overnight. Following one wash with PBS, cells were treated with Dynasore (80µM, Selleckchem, catalog# S8047), 25mM ammonium chloride (Sigma-Aldrich catalog, # 254134) or 0.2% DMSO (Sigma-Aldrich, catalog# D8418) in DMEM for 30 minutes at 37°C.

### Transfections

HEK293T cells were co-transfected with 0.5µg of pLVX-Neo1 and 0.5µg of pLVX-Epsin 1- or pLVX-GFP-v5-TurboID in Opti-MEM with Fugene (Promega, catalog# E2311) as per manufacturer’s instructions. Cells were incubated with DNA and Fugene mixture for 48 hours.

A549, MRC-5 and MRC-5 hTERT cells were reverse transfected with siRNAs (Qiagen, S3 Table) in OptiMEM (Life Technologies, catalog# 13778150) using Lipofectamine RNAiMax (Invitrogen, catalog# 13778150) as per manufacturer’s instructions. Cells were incubated with siRNAs and lipofectamine mixture for 48 hours.

### Overrepresentation analysis

Potential epsin 1 interactors were analyzed with g:Profiler using a Benjamini-Hochberg FDR of 0.05 [75]. The highest and lowest –log p_adj_ values for enriched terms within each category were used to generate a scale bar to represent data in heat map format.

### Protein interaction network

A network depicting high confidence functional and physical interactions (score > 0.7) of potential epsin 1 interactors from IAV-infected samples was generated with stringApp in Cytoscape v3.10.2. Known high confidence functional and physical interactors of epsin 1 were retrieved from the STRING database (https://string-db.org/). Proviral host factors enriched upon IAV-infection were identified by querying Neo1 and potential epsin 1 interactors detected only upon infection in the IAV Meta Database [34] (https://metascape.org/IAV/).

### Analysis of transmembrane regions in proteins

The presence of transmembrane domains was assessed with DeepTMHMM [35]. Potential epsin 1 interactors from IAV-infected samples with a Plasma Membrane GO term annotation were considered as possible receptor candidates and subjected to the analysis. To do so, sequences for their main human isoform were retrieved from UniProt and uploaded into DeepTMHMM.

### Neo1 expression in primary bronchial epithelial cells

Data from [39] was analyzed to assess the expression of Neo1, Nectin2, EGFR and GRM2 in human primary bronchial epithelial cells (BEpCs). As done previously [39], the single-cell transcriptome data from uninfected BEpCs was grouped into goblet, basal, club, basal differentiating into secretory (BdiS), ciliated, suprabasal or undefined cell clusters using the Seurat framework (v4.3) based on the expression of known cell subtype marker genes. The SCTransform-normalized expression of selected transcripts therein was analyzed in RStudio to generate the RidgePlots.

### A549 cell surface proteome

Data from the proteomics results in [38] was analyzed to evaluate protein abundance at the A549 cell surface. Proteins with an ms1 intensity > 0 in both samples, a total number of unique peptide to spectrum matches ≥ 2, a transmembrane domain number ≥ 1 and a plasma membrane or undefined membrane localization were considered for the analysis. The average ms1 intensity for each protein was calculated from the normalized ms1 intensity across both replicates and was divided by the mean normalized ms1 intensity of all proteins. A549 cell surface proteins were ranked according to their relative abundance.

### A/WSN/33 infection

To assess multicycle growth, siRNA-treated A549 cells were washed once with PBS and inoculated with A/WSN/33 at an MOI of 0.01 in PBSi for 1 hour at 37°C. After removing the inoculum, cells were washed once with PBS and maintained in piDMEM supplemented with 1µg/mL TPCK-trypsin at 37°C. Supernatant was collected at the indicated times post-infection and viral titers assessed by plaque assay in MDCK cells. Each biological replicate was performed in technical duplicates. Data was analyzed with GraphPad Prism.

To assess viral nucleoprotein expression, inhibitor-treated A549 LV-EPN1-v5-TurboID cells were washed once with PBS, cooled on ice for 10 minutes and inoculated with A/WSN/33 at an MOI of 10 in PBSi for 1 hour on ice. After inoculum removal, cells were washed once with PBS and maintained in DMEM supplemented with the corresponding inhibitors for 1 hour at 37°C. At this time, cells were washed once with PBS and maintained in piDMEM for 3.5hs at 37°C prior to fixation with 3.7% paraformaldehyde (PFA).

To assess IAV and Neo1 co-localization in non-permeabilized and in permeabilized samples, A549 LV Neo1 cells were grown on coverslips overnight at 37°C and washed once with PBS. For the former, cells were inoculated with A/WSN/33 at an MOI of 100 in DMEM and maintained for 2 or 5 minutes at 37°C. For the latter, cells were pre-cooled on ice and inoculated with A/WSN/33 at an MOI of 25 in DMEM for 1 hour on ice, washed twice with cold PBS and maintained in DMEM for 0, 5, 15 or 30 minutes at 37°C. After inoculum or DMEM removal, cells were rinsed three times with PBS and fixed with 3.7% PFA.

### IAV *Renilla* infection

The infection was done as in [45]. siRNA-treated A549, MRC-5 or MRC-5 hTERT cells were washed once with PBS and inoculated with Renilla-encoding IAVs at the indicated MOI in PBSi for 1 hour at 37°C. Following inoculum removal, cells were washed once with PBS and maintained in piDMEM supplemented only with 6µM EnduRen Live Cell Substrate (Promega, catalog# E6482) or also with 25mM NH4Cl (H1N1 in A549 cells) at 37°C. Luminescence was sampled at the indicated times post-infection using a Perkin Elmer plate reader. Mock-infected cells were used to determine background luminescence and the AUC above it was calculated with GraphPad Prism.

### VSV infection

A549 LV Neo1 cells grown on coverslips were washed once with PBS, pre-cooled on ice and inoculated with VSV at an MOI of 150 in PBSi for 1 hour on ice. Following inoculum removal, cells were washed twice with PBS and maintained in DMEM for 0 or 15 minutes at 37°C prior to fixation with 3.7% PFA.

### VSV-GFP and PIV5-GFP infections

Inhibitor or siRNA-treated A549 cells were washed once with PBS and inoculated with VSV-GFP or PIV5-GFP at an MOI of 3 in PBSi or DMEM supplemented with the corresponding inhibitors for 1 hour at 37°C. Following inoculum removal, cells were washed once with PBS and maintained in piDMEM at 37°C. GFP expression and cell confluence were evaluated at the indicated times post-infection with an Incucyte S3 Live-Cell Analysis Instrument (Sartorius). Total Green Integrated Intensity (Green Calibrated Unit x um2/image) values were calculated with the Incucyte software and normalized to cell confluence. Uninfected cells were utilized to determine background signal and the area under the curve (AUC) above background signal was calculated with GraphPad Prism.

### BlaM1 VLP infection

BlaM1 VLPs pseudotyped with the HA and NA of A/WSN/33 were generated and the infection performed as previously described [63]. In brief, siRNA-treated MRC-5 cells were washed once with PBS and infected with the BlaM1 VLPs in OptiMEM supplemented with 2% FCS and 0.1µg/mL DEAE-Dextran for 4 hours at 37°C. Subsequently, cells were detached by trypsinization, collected and incubated with β-lactamase substrate CCF2-AM (ThermoFisher Scientific, catalog# K1032) and LIVE/DEAD Fixable Near-IR Dead Cell Stain (ThermoFisher Scientific, catalog# L10119) in OptiMEM for 45 minutes at 37°C. Samples were analyzed on a BD FACSymphony S1 flow cytometer. Following exclusion of dead cells, entry positive cells were determined by gating on those with cleaved CCF2-AM using FlowJo v10.8.1.

### Cell viability

Cell viability in inhibitor or siRNA-treated A549 cells was evaluated with a CellTiter-Glo Luminescent Cell Viability Assay kit (Promega, catalog# G7571) as per manufacturer’s instructions. The signal was recorded on a Perkin Elmer plate reader. Data was analyzed with GraphPad Prism and is denoted relative to the control treatments (siSCR or DMSO).

### RT-qPCR

The efficacy of siRNA knockdown of Neo1 expression was assessed via RT-qPCR. RNA was extracted from siRNA-treated A549 cells using a ReliaPrep RNA Cell Miniprep kit (Promega, catalog# Z6011). cDNA was generated using oligo(DT) primers and the SuperScript IV First-Strand Synthesis System (ThermoFisher Scientific, catalog# 18091050) as per manufacturer’s instructions. RT-qPCR was performed with diluted cDNA, primers described below and PowerTrack SYBR Green Master Mix (ThermoFisher Scientific, catalog# A46012) using a 7300 Real-Time PCR System (Applied Biosystems). GADPH was included as an internal control. The sequences of all primers utilized can be found in S3 Table. Changes in relative Neo1 expression were calculated using the 2^–∆∆Ct^ method [47] and are shown relative to siSCR-treated cells.

### AFM

AFM tips were functionalized with IAV attached using an aldehyde-PEG_24_-NHS linker, following a previously described protocol [40,41]. Functionalized AFM tips were stored in buffer (150 mM NaCl, 15 mM MgCl_2_ and 10 mM Tris) at 4 °C for a maximum of 24 hours. Gold-coated surfaces were functionalized with Neo1 (0.1 mg mL − 1, Biotechne catalog# 8607-NE) using NHS/EDC chemistry, as previously described [76]. Surfaces were kept hydrated and used immediately after preparation. Force-distance (FD) curve experiments were performed using a ForceRobot-400 (JPK, Berlin) AFM, equipped with virus-functionalized MCST-D cantilevers (Bruker, Santa Barbara). The spring constant of the cantilever was calculated using the thermal noise method, obtaining a spring constant of approximately 0.04 N/m. For all FD experiments, a ramp size of 500 nm was used, with an approach velocity of 1 µm/s and a maximum applied force of 500 pN. Binding probability assays were performed in DPBS. The specificity of the interaction was confirmed by blocking experiments, measuring the binding probability before and after incubation of the model surface with anti-Neo1 for 15 minutes at a concentration of 100 µg/ml. Measurement of k_on_ was conducted by varying the hold time (0, 50, 100, 250, 500, and 1000 ms) with a retraction velocity of 1 µm/s. An exponential decay model was fitted to the binding probability to obtain the decay constant of the interaction of k_on_ [42]. Measurement of k_off_ was conducted by varying the retraction velocities (0.1, 0.3, 1, 3, and 10 µm/s) and no surface delay. Selected FD curves were fitted with the worm-like chain model to obtain the loading rate and the breaking force. The breaking force for each loading rate segment was determined using a force histogram fitted with a gaussian curve distribution (S3 Fig). The Bell-Evans model was fitted to the breaking force at different loading rates to obtain k_off_, x_u_, [43,77] and the dissociation constant K_D_ [42].

To evaluate the effect of sialic acid and glycosylation on binding, binding frequency measurements were performed by measuring the binding probability of IAV with Neo1 in DPBS, compared to a surface incubated with 1 mM of free sialic acid (N-acetylneuraminic acid, Merk, catalog# 19023), and a surface incubated with 500 units/µl of N-glycosidase (PNGase F, Biolabs catalog# P0704S).

### Immunofluorescence

The target cell lines were grown on coverslips at 37°C and treated or IAV-infected as indicated. After three washes with PBS, cells were fixed by incubation with 3.7% PFA in PBS for 15 minutes. For experiments under non-permeabilized conditions, samples were incubated with 5µg/mL Wheat Germ Agglutinin-Alexa Fluor 488 (ThermoFisher Scientific, catalog# W11261) for 15 minutes in PBS and blocked with 2% BSA in PBS for 1 hour. For experiments to validate construct expression, samples were permeabilized and blocked by incubation with 0.5% Triton X-100 in PBS for 5 minutes and 2% FBS in PBS for 30 minutes, respectively. For experiments to assess viral nucleoprotein expression and to evaluate Neo1, clathrin and VSV or IAV co-localization, samples were both permeablized and blocked by incubation with confocal buffer (PBS supplemented with 50mM ammonium chloride (Sigma-Aldrich, catalog# 254134), 0.1% saponin (Sigma-Aldrich, catalog# 47036) and 2% BSA (Sigma-Aldrich, catalog# A7906)) for 1 hour. Samples were incubated with primary antibodies and secondary antibodies diluted in their blocking buffer for 1 hour. For the assessment of Neo1, VSV or IAV and clathrin co-localization, samples were incubated in a subsequent step with 1:500 anti-clathrin heavy chain-Alexa Fluor 488 (ThermoFisher Scientific, catalog# MA1–065-A488) in confocal buffer for 1 hour. Samples were mounted with ProLong Gold Antifade Mountant (ThermoFisher Scientific, catalog# P36930) and imaged with a confocal SP8 microscope using a 63x objective (Leica) or DMIL LED fluorescent microscope (Leica). Samples imaged with the latter were also assessed with a PerkinElmer plate reader to quantify the Alexa Fluor 488 signal intensity, shown as Relative Fluorescence Units (RFU), using a FITC mirror. The background signal was determined using empty wells. The following primary antibodies were used: 1:250 anti-Neo1 (Abcam, catalog# ab183511), 1:10 anti-IAV HA (WCL50), 1:50 anti-Epsin 1 (Santa Cruz, catalog# sc-55556), 1:1000 anti-v5 (Bio-Rad, catalog# MCA1360), 1:5 anti-IAV NP (HB65, catalog# H16-L10-4R5, ATCC), 1:500 anti-VSV-G (8G5F11, Kerafast, catalog# EB0010). In conjunction with 1:1000 DAPI (Sigma-Aldrich, catalog# 10236276001), the following secondary antibodies were used at a 1:500 dilution: Donkey anti-Mouse IgG (H + L) Highly Cross-Adsorbed Secondary Antibody, Alexa Fluor 546 (ThermoFisher Scientific, catalog# A10036), Goat anti-Rabbit IgG (H + L) Highly Cross-Adsorbed Secondary Antibody, Alexa Fluor 633 (ThermoFisher Scientific, catalog# A21071), Donkey anti-Mouse IgG (H + L) Highly Cross-Adsorbed Secondary Antibody, Alexa Fluor 555 (ThermoFisher Scientific, catalog# A31570), Donkey anti-Mouse IgG (H + L) Highly Cross-Adsorbed Secondary Antibody, Alexa Fluor 488 (ThermoFisher Scientific, catalog# A21202).

### Image analysis

Confocal microscopy images from experiments that assessed Neo1 and IAV or VSV co-localization in permeabilized or non-permeabilized samples were deconvolved using Huygens Professional v23.04 (Scientific Volume Imaging, The Netherlands, http://svi.nl). The deconvolved images were visualized in Imaris 10.2.0 to generate snapshots of Z-stack images and orthogonal sections. An intensity-based co-localization channel was generated to denote co-localization between Neo1 and IAV or VSV by sampling the average intensity for each channel to establish a minimum intensity threshold.

Images from experiments that assessed Neo1, clathrin and IAV or VSV co-localization were further analyzed in Imaris 10.2.0 akin to [78] to obtain a per cell quantification of co-localization between IAV or VSV and clathrin alone or in conjunction with Neo1. In brief, DAPI and Neo1 signal were utilized to render cell nuclei and cytoplasm, respectively, to segment for cells using the Cell Module. Next, clathrin and IAV or VSV were rendered as spots using the Spot Module (clathrin dimensions: xy 0.35µm, z 0.6µm with a quality above 4.6 or 8.5; IAV dimensions: xy 0.35µm, z 0.5µm with a quality above 12; VSV dimensions: xy 0.4µm, z 0.8µm with a quality above 12). To render Neo1 co-localized IAVs or VSVs, Co-loc spots were generated from the co-localization channel using the Spot Module (IAV Co-loc dimensions: xy 0.35µm, z 0.5µm with a quality above 4.5; VSV Co-loc dimensions: xy 0.4µm, z 0.8µm with a quality above 4.5). IAV and VSV spots at a distance less than 0.35µm or 0.4µm, respectively, to clathrin or Co-loc spots were deemed to co-localize. All spots were incorporated into cells as Vesicle objects to obtain per cell counts. To exclude outlier effects, only IAV- or VSV-infected cells containing at least 13 and less than 100 IAVs or VSVs were considered. At least 3 cells satisfying these conditions were obtained for each replicate.

## Supporting information

S1 Table List of proteins identified by proximity labelling analyzed with SAINTexpress. (XLSX)

S2 Table List of terms from overrepresentation analysis. (XLSX)

S3 Table Primers and siRNAs used in this study. (XLSX)

S1 Fig Dynasore or ammonium chloride pre-treatment reduces replication of VSV-GFP but not PIV5-GFP. A549 LV-EPN1-v5-TurboID cells were treated with DMSO (0.2%), NH_4_Cl (25mM) or Dynasore (80µM) for 30 minutes and then inoculated with VSV-GFP (**A**) or PIV5-GFP (**C**) at an MOI of 3 in presence of the inhibitors. The GFP signal was recorded at the indicated times post-infection and the total integrated green signal intensity normalized to overall cell confluence. The dashed line indicates the highest normalized intensity value obtained in uninfected cells. **B, D)** The area under the curve (AUC) above background was calculated for the infections in **A** and **C**, respectively, until 9 and 21 hours post-infection. Statistical significance was inferred by two-tailed, one sample t-test with a theoretical mean of 100. *p < 0.05, **p < 0.01, ns = not significant. **A-D)** Data are means ± standard deviation from N = 3 independent experiments.
(TIFF)

S2 Fig Assessment of Neo1 expression in A549 and primary human bronchial epithelial cells. **A)** Proteins identified in cell surface proteome mapping experiments in [38] were analyzed to determine the relative abundance of Neo1, Nectin2 and EGFR at the A549 cell surface. Akin to [38], proteins were filtered for an ms1 intensity value greater than 0 in both replicates and at least two unique peptides matching the spectra. Cell surface proteins were then determined by screening for the presence of at least one annotated transmembrane domain and a Uniprot plasma membrane or undefined membrane localization. Their normalized ms1 intensity across both replicates was averaged, divided by the mean intensity of all surface proteins identified in A549 cells and the resulting value multiplied by 100 to aid in visualization. Proteins are ordered from most to least abundant. The abundance ranking of EGFR, Nectin2 and Neo1 is denoted. **B)** The single cell transcriptome data from primary human bronchial epithelial cells (BEpCs) of donor AB079 from [39] was analyzed to assess expression of Neo1, Nectin 2, GRM2 (mGluR2) and EGFR via Seurat. In the original experiment, BEpCs were processed for single-cell sequencing and the transcriptomes of ~7,000 cells were obtained. After cluster identification, cell subtype allocation was performed on the basis of expression of canonical markers. RidgePlots depicting SCTtransform-normalized expression of Neo1, Nectin 2, GRM2 (mGluR2) and EGFR within each cell subtype cluster are shown. Values reflect deviation from expected expression under a regularized negative binomial model.
(TIFF)

S3 Fig Extraction of rupture forces of IAV and Neo1. Loading rates and forces were extracted from force-distance curves and divided into loading rate segments. For each segment, a histogram of the rupture force distribution is plotted and fitted with multipeak Gaussian peaks. The maximum force value is indicated for each fitted peak.
(TIFF)

S4 Fig Additional examples of Neo1 co-localization with incoming virus at the cell surface. **A)** A549 LV-Neo1 cells were inoculated with A/WSN/33 at an MOI of 100 and maintained for 2 or 5 minutes-post infection at 37°C prior to fixation. Non-permeabilized samples were incubated with WGA-AF488 lectin, anti-Neo1 and anti-IAV HA antibodies and their expression evaluated via confocal microscopy, 63x objective. Images are Z-stack slices that depict sialic acid (Sia, red), Neo1 (magenta), IAV HA (green) and cell nuclei (DAPI, blue). White arrows indicate Neo1 and IAV co-localization. **B)** Orthogonal sections of A549 LV-Neo1 cells inoculated with A/WSN/33 maintained for 2 or 5 minutes post-infection shown in **A** (enclosed by a dashed white box). Neo1 and IAV HA co-localization was additionally analyzed via intensity-based co-localization (cyan). Images are representative of N = 2 independent experiments. The scale bar represents 4 and 2µm for **A** and **B**, respectively.
(TIFF)

S5 Fig Quantification of Neo1 expression, clathrin, IAV and VSV spots, and VSV co-localization with clathrin and Neo1 in virus-infected A549 cells at early times post-infection. Images from Fig 6 were analyzed with Imaris to determine Neo1 Mean Fluorescent Intensity (MFI) per image, amount of CHC spots and amount of IAV or VSV spots per cell upon infection with IAV (**A-C**) or VSV (**D-F**). **G, H)** A549 LV-Neo1 cells were inoculated with VSV at an MOI of 150 and, following cold-binding for 1 hour, incubated for 0 or 15 minutes at 37°C. Samples were fixed, permeabilized and probed with anti-Neo1, CHC and VSV-G antibodies. Their expression was assessed by confocal microscopy and the deconvolved images processed as in Fig 6C and 6D to generate **G** and **H**, respectively. The distance to determine co-localization was 0.4µm to account for the larger VSV diameter. Statistical significance was inferred by unpaired two-tailed Mann-Whitney test. p**** < 0.0001. **A-H**) Data are means ± standard deviation from N = 3–4 (**A-C**) or N = 3 (**D-H**) independent experiments. Each dot represents an individual image (**A**, **D**) or cell (**B-C**, **E-H**).
(TIFF)
