# Supplementary figures and images for: Proximity labelling of internalizing influenza A viruses reveals a role for neogenin in virus uptake
Source: PLoS Pathog. 2025 Jul 7;21(7):e1013338. doi: 10.1371/journal.ppat.1013338 (PMC12258580; doi:10.1371/journal.ppat.1013338)

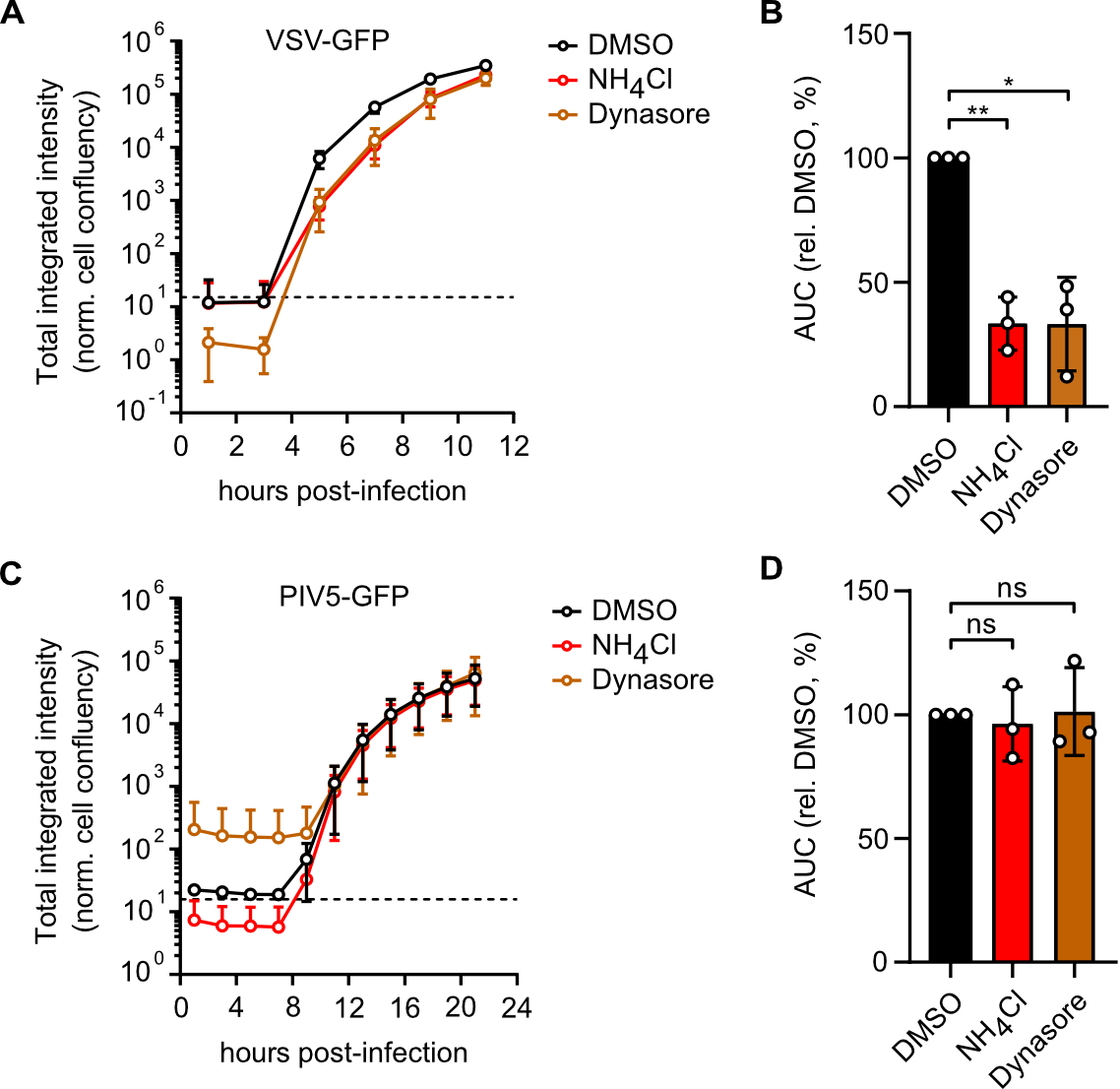

Supplement: S1 Fig — A549 LV-EPN1-v5-TurboID cells were treated with DMSO (0.2%), NH4Cl (25mM) or Dynasore (80µM) for 30 minutes and then inoculated with VSV-GFP (A) or PIV5-GFP (C) at an MOI of 3 in presence of the inhibitors. The GFP signal was recorded at the indicated times post-infection and the total integrated green signal intensity normalized to overall cell confluence. The dashed line indicates the highest normalized intensity value obtained in uninfected cells. B, D) The area under the curve (AUC) above background was calculated for the infections in A and C, respectively, until 9 and 21 hours post-infection. Statistical significance was inferred by two-tailed, one sample t-test with a theoretical mean of 100. *p < 0.05, **p < 0.01, ns = not significant. A-D) Data are means ± standard deviation from N = 3 independent experiments. (TIFF) [file ppat.1013338.s004.tiff]

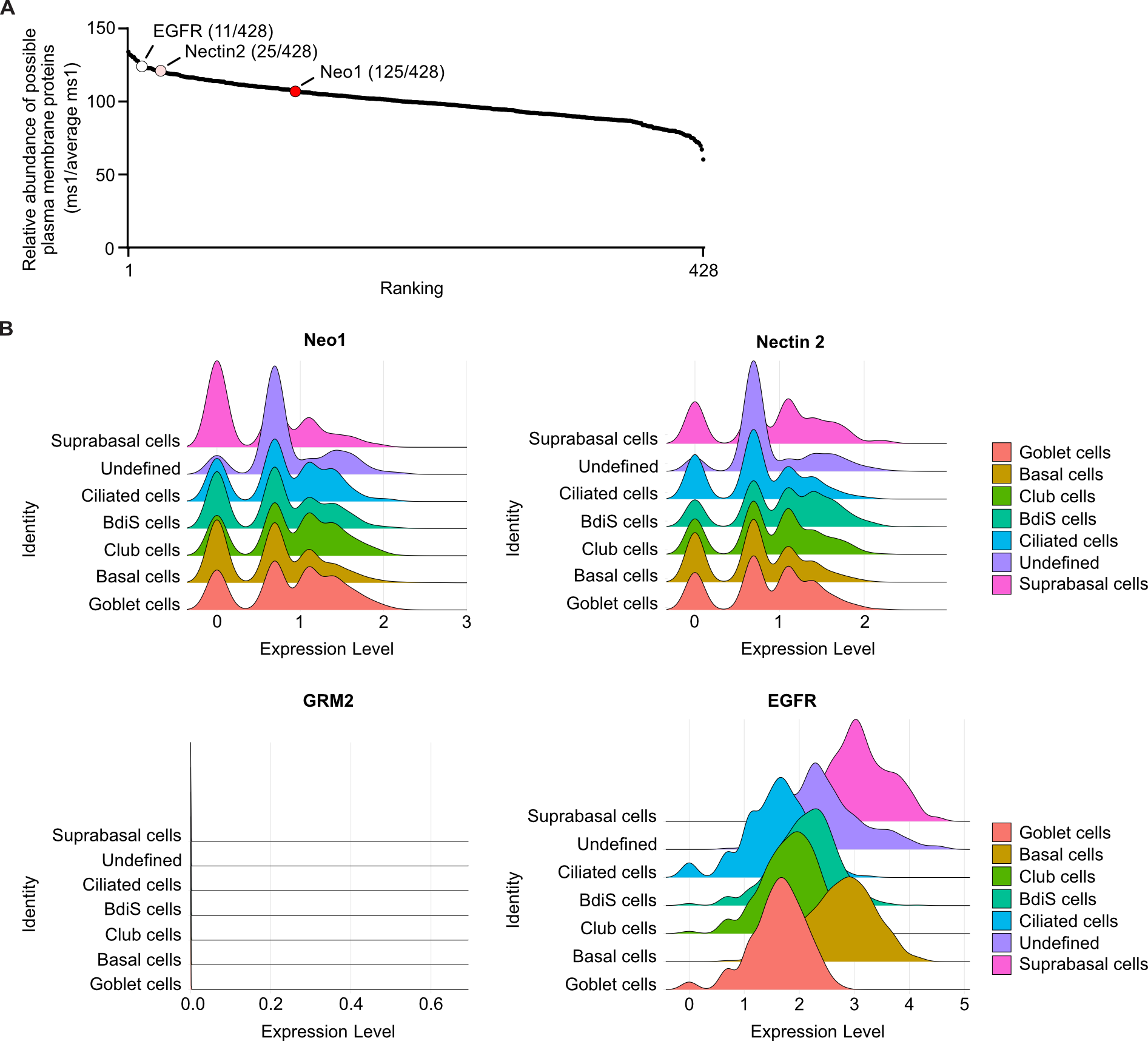

Supplement: S2 Fig — A) Proteins identified in cell surface proteome mapping experiments in [38] were analyzed to determine the relative abundance of Neo1, Nectin2 and EGFR at the A549 cell surface. Akin to [38], proteins were filtered for an ms1 intensity value greater than 0 in both replicates and at least two unique peptides matching the spectra. Cell surface proteins were then determined by screening for the presence of at least one annotated transmembrane domain and a Uniprot plasma membrane or undefined membrane localization. Their normalized ms1 intensity across both replicates was averaged, divided by the mean intensity of all surface proteins identified in A549 cells and the resulting value multiplied by 100 to aid in visualization. Proteins are ordered from most to least abundant. The abundance ranking of EGFR, Nectin2 and Neo1 is denoted. B) The single cell transcriptome data from primary human bronchial epithelial cells (BEpCs) of donor AB079 from [39] was analyzed to assess expression of Neo1, Nectin 2, GRM2 (mGluR2) and EGFR via Seurat. In the original experiment, BEpCs were processed for single-cell sequencing and the transcriptomes of ~7,000 cells were obtained. After cluster identification, cell subtype allocation was performed on the basis of expression of canonical markers. RidgePlots depicting SCTtransform-normalized expression of Neo1, Nectin 2, GRM2 (mGluR2) and EGFR within each cell subtype cluster are shown. Values reflect deviation from expected expression under a regularized negative binomial model. (TIFF) [file ppat.1013338.s005.tiff]

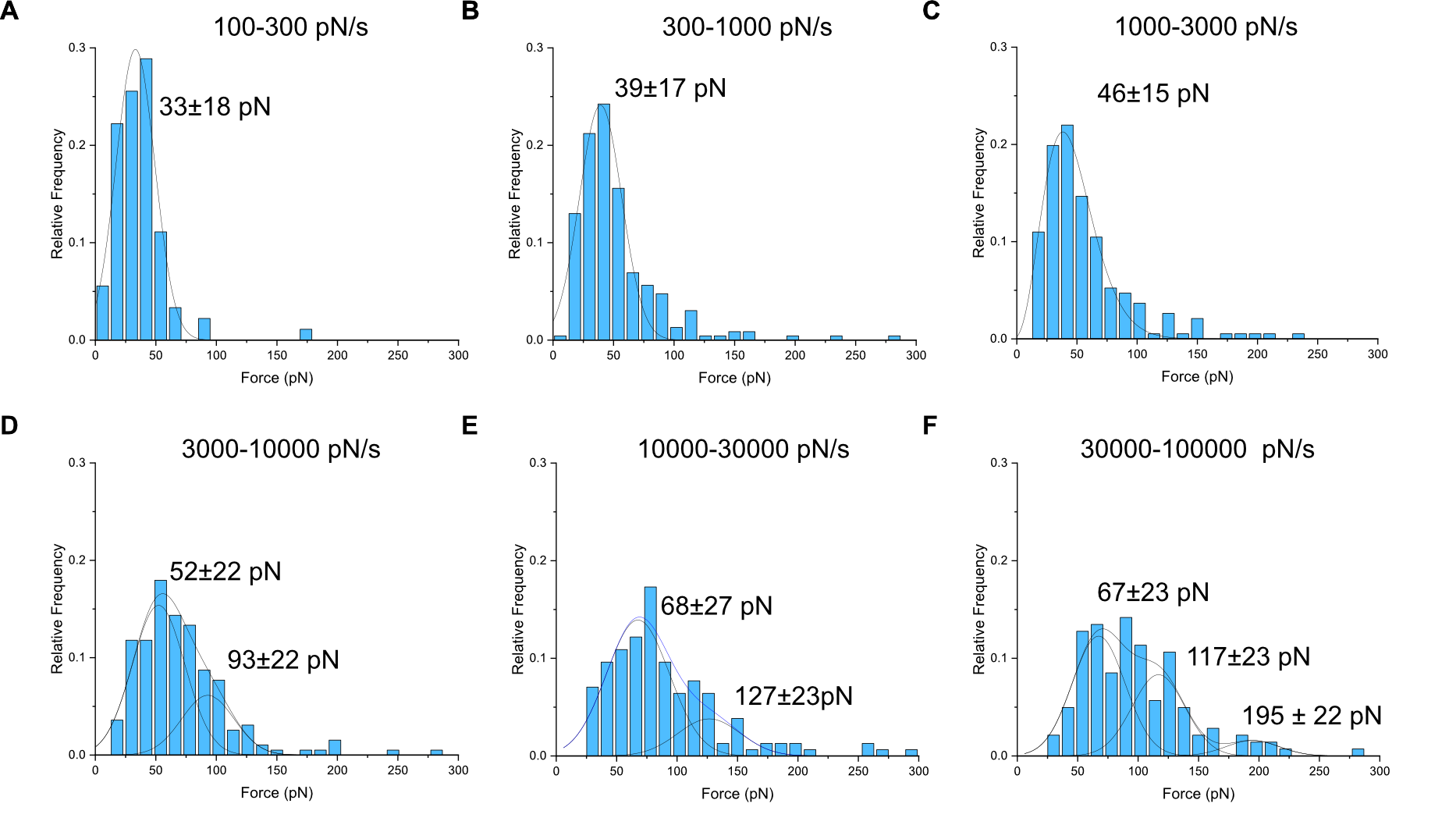

Supplement: S3 Fig — Loading rates and forces were extracted from force-distance curves and divided into loading rate segments. For each segment, a histogram of the rupture force distribution is plotted and fitted with multipeak Gaussian peaks. The maximum force value is indicated for each fitted peak. (TIFF) [file ppat.1013338.s006.tiff]

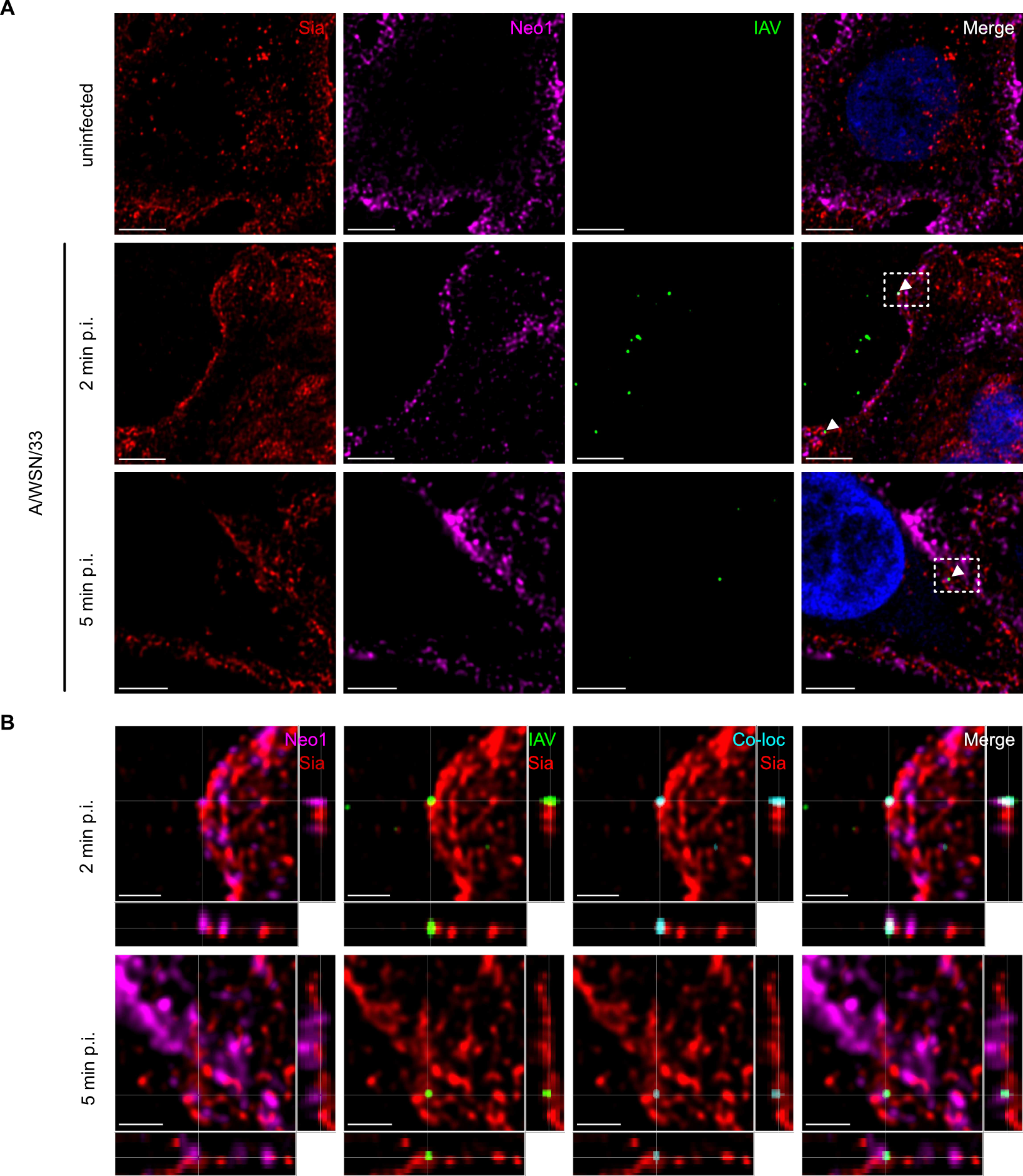

Supplement: S4 Fig — A) A549 LV-Neo1 cells were inoculated with A/WSN/33 at an MOI of 100 and maintained for 2 or 5 minutes-post infection at 37°C prior to fixation. Non-permeabilized samples were incubated with WGA-AF488 lectin, anti-Neo1 and anti-IAV HA antibodies and their expression evaluated via confocal microscopy, 63x objective. Images are Z-stack slices that depict sialic acid (Sia, red), Neo1 (magenta), IAV HA (green) and cell nuclei (DAPI, blue). White arrows indicate Neo1 and IAV co-localization. B) Orthogonal sections of A549 LV-Neo1 cells inoculated with A/WSN/33 maintained for 2 or 5 minutes post-infection shown in A (enclosed by a dashed white box). Neo1 and IAV HA co-localization was additionally analyzed via intensity-based co-localization (cyan). Images are representative of N = 2 independent experiments. The scale bar represents 4 and 2µm for A and B, respectively. (TIFF) [file ppat.1013338.s007.tiff]

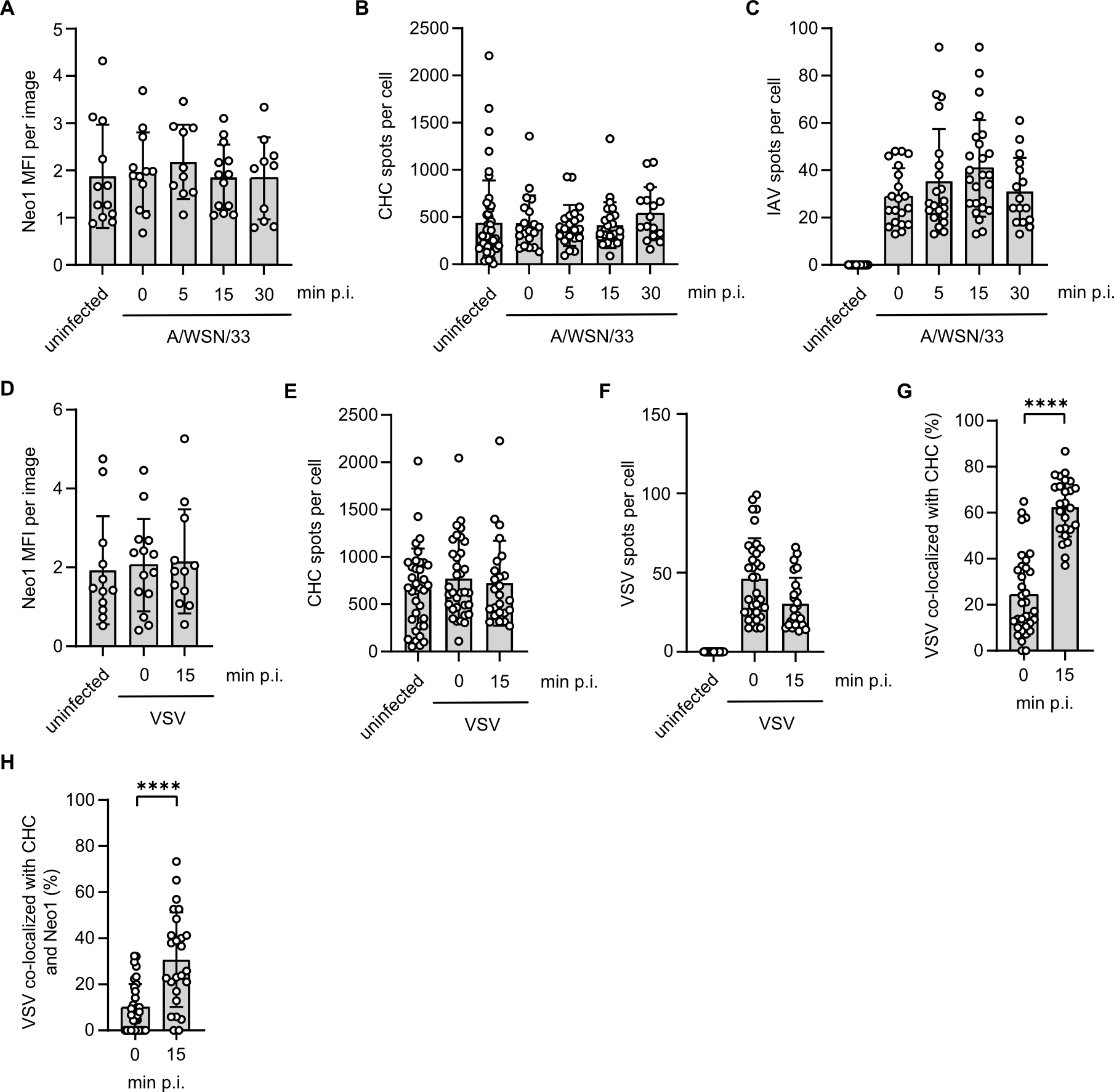

Supplement: S5 Fig — Images from Fig 6 were analyzed with Imaris to determine Neo1 Mean Fluorescent Intensity (MFI) per image, amount of CHC spots and amount of IAV or VSV spots per cell upon infection with IAV (A-C) or VSV (D-F). G, H) A549 LV-Neo1 cells were inoculated with VSV at an MOI of 150 and, following cold-binding for 1 hour, incubated for 0 or 15 minutes at 37°C. Samples were fixed, permeabilized and probed with anti-Neo1, CHC and VSV-G antibodies. Their expression was assessed by confocal microscopy and the deconvolved images processed as in Fig 6C and 6D to generate G and H, respectively. The distance to determine co-localization was 0.4µm to account for the larger VSV diameter. Statistical significance was inferred by unpaired two-tailed Mann-Whitney test. p**** < 0.0001. A-H) Data are means ± standard deviation from N = 3–4 (A-C) or N = 3 (D-H) independent experiments. Each dot represents an individual image (A, D) or cell (B-C, E-H). (TIFF) [file ppat.1013338.s008.tiff]
